# The Outcomes of Surgical Pulmonary Embolectomy for Pulmonary Embolism: A Meta-Analysis

**DOI:** 10.3390/jcm13144076

**Published:** 2024-07-12

**Authors:** Mohamed Rahouma, Shaikha Al-Thani, Haitham Salem, Alzahraa Mahmoud, Sherif Khairallah, David Shenouda, Batool Sultan, Laila Khalil, Mohammad Alomari, Mostafa Ali, Ian A. Makey, John C. Haney, Stephanie Mick, Magdy M. El-Sayed Ahmed

**Affiliations:** 1 Cardiothoracic Surgery Department, Weill Cornell Medicine, New York, NY 10065, USA; sma9023@med.cornell.edu (S.A.-T.); smk4005@med.cornell.edu (S.K.); slmick@med.cornell.edu (S.M.); 2 Surgical Oncology Department, National Cancer Institute, Cairo University, Cairo 11796, Egypt; 3 Ain Shams University Hospital, Ain Shams University, Cairo 11517, Egypt; haithamsalem.md@gmail.com; 4 Faculty of Medicine, Beni Suef University, Beni Suef 2721562, Egypt; alzahraamah@gmail.com; 5 New York Institute of Technology, New York, NY 10023, USA; shenoudadavid17@gmail.com; 6 Rak Medical and Health Sciences University, Ras al Khaimah 11172, United Arab Emirates; batoolsultan77@gmail.com; 7 Weill Cornell Medicine, Doha 24144, Qatar; ltk4001@qatar-med.cornell.edu; 8 Cardiothoracic Surgery Department, Mayo Clinic, Jacksonville, FL 32224, USA; alomari.mohammad@mayo.edu (M.A.); ali.mostafa@mayo.edu (M.A.); makey.ian@mayo.edu (I.A.M.); haney.john@mayo.edu (J.C.H.); 9 Surgery Department, Faculty of Medicine, Zagazig University, Zagazig 44519, Egypt

**Keywords:** surgical pulmonary embolectomy, pulmonary embolism, hospital mortality, pulmonary bleeding, thrombolysis

## Abstract

**Objectives:** The purpose of this study is to assess the efficacy, short- and long-term cardiovascular and non-cardiovascular mortalities and postoperative morbidities of surgical pulmonary embolectomy (SPE) for patients with massive or submassive pulmonary embolism. Methods: A comprehensive literature review was performed to identify articles reporting SPE for pulmonary embolism. The outcomes included in-hospital and long-term mortality in addition to postoperative morbidities. The random effect inverse variance method was used. Cumulative meta-analysis, leave-one-out sensitivity analysis, subgroup analysis and meta-regression were performed. **Results:** Among the 1949 searched studies in our systematic literature search, 78 studies met our inclusion criteria, including 6859 cases. The mean age ranged from 42 to 65 years. The percentage of males ranged from 25.6% to 86.7%. The median rate of preoperative cardiac arrest was 27.6%. The percentage of contraindications to preoperative systemic thrombolysis was 30.4%. The preoperative systemic thrombolysis use was 11.5%. The in-hospital mortality was estimated to be 21.96% (95% CI: 19.21–24.98); in-hospital mortality from direct cardiovascular causes was estimated to be 16.05% (95% CI: 12.95–19.73). With a weighted median follow-up of 3.05 years, the late cardiovascular and non-cardiovascular mortality incidence rates were 0.39 and 0.90 per person-year, respectively. The incidence of pulmonary bleeding, gastrointestinal bleeding, surgical site bleeding, non-surgical site bleeding and wound complications was 0.62%, 4.70%, 4.84%, 5.80% and 7.2%, respectively. Cumulative meta-analysis showed a decline in hospital mortality for SPE from 42.86% in 1965 to 20.56% in 2024. Meta-regression revealed that the publication year and male sex were associated with lower in-hospital mortality, while preoperative cardiac arrest, the need for inotropes or vasopressors and preoperative mechanical ventilation were associated with higher in-hospital mortality. **Conclusions**: This study demonstrates acceptable perioperative mortality rates and late cardiovascular and non-cardiovascular mortality in patients who undergo SPE for massive or submassive pulmonary embolism.

## 1. Introduction

Venous thromboembolisms, such as pulmonary embolisms (PEs), are the third most common cardiovascular (CV) syndrome, with increasing incidence in the aging population [1,2]. PE has been reported in the literature to have a high mortality rate [3]. The clinical presentation of PE is often non-specific and can range from incidental findings on a computed tomographic chest scan with no clinical symptoms to patients presenting with hemodynamic instability, defined as individuals who are hypotensive needing pressor support and develop end organ hypoperfusion, and sudden death. Nevertheless, presentation in extremes accounts for only 5% of PE cases. 

The primary mode of treatment for acute PE is anticoagulation (1). According to the European Society of Cardiology guidelines, surgical pulmonary embolectomy (SPE) for the treatment of PE should be reserved for individuals who deteriorate hemodynamically while being on rescue thrombolytic therapies, for those with contraindications for thrombolytic therapies or for failed catheter-directed thrombolysis [1,2]. SPE usually included the performance of cardiopulmonary bypass (CPB), and the literature has demonstrated varying outcomes following surgical intervention [3]. Therefore, we performed a systematic review and meta-analysis to assess the efficacy and short- and long-term CV and non-CV mortalities for patients that present with PE. 

## 2. Materials and Methods

This meta-analysis was performed in concordance with the Preferred Reporting Items for Systematic Reviews and Meta-Analyses (PRISMA) statement [4] and AMSTAR (A MeaSurement Tool to Assess systemic Reviews) Guideline.

### 2.1. Search Strategy 

On 14 March 2024, the PubMed and Scopus databases were systematically searched for publications on SPE. The search terms in subject headings and main keywords included the following: “Pulmonary Embolectomy”, “surgical embolectomy”, “surgical pulmonary embolectomy”, “surgical intervention”, and “pulmonary embolism”. This review was registered with the PROSPERO register of systematic reviews (ID: 542752). There was no individual patient involvement in this study; as such, research ethics board approval was not required. 

### 2.2. Study Selection and Inclusion Criteria

Two investigators (HS, SA) independently performed data extraction. Database searches were conducted, and article de-duplication and screening were performed by these two reviewers. A third independent reviewer (MR) confirmed the adequacy of the studies based on the predefined inclusion and exclusion criteria. Articles were included if they were in full-text English on human subjects that included five or more patients with reported CV or non-CS mortality or morbidity outcomes following SPE. We included studies with the largest sample size and the most comprehensive follow-up period for each outcome of cumulative or longitudinal results in more than one publication. Studies were excluded if they were in a non-English language, did not include SPE, did not specify the number or proportion of mortality or morbidity or had a small case series with less than 5 patients. 

The full article text of the screened studies was retrieved for the second round of eligibility screening. Prior meta-analyses and systematic reviews were searched to confirm the inclusion of all eligible studies (i.e., backward snowballing). A PRISMA flow diagram illustrating the study selection process is available in the Supplementary Materials (Supplementary Figure S1). The Newcastle–Ottawa scale (NOS) for assessing the quality of Cohort Studies was used for the critical appraisal of eligible studies. Studies with scores of six or more were included [5].

### 2.3. Clinical Outcomes/Definitions

The primary outcome of interest was SPE hospital mortality. Secondary outcomes included CV and non-CV mortality, postoperative pulmonary bleeding, gastrointestinal (GI) bleeding, surgical site bleeding, non-surgical site bleeding and wound complication. 

Subgroup analysis for the primary outcome was conducted based on continents.

### 2.4. Data Extraction and Statistical Analysis

Extracted variables included the following: study name, publication year, study design, mean age, percentage of males, mean follow-up in years, percentage of individuals with a contraindication to systemic thrombolytic therapy, percent of preoperative cardiac arrest, preoperative mechanical ventilation, percent of individuals that underwent CPB or extracorporeal membrane oxygenation (ECMO) support, percent of right ventricular (RV) dysfunction, the need for inotropes or vasopressors, systemic thrombolysis, use of myocardial protective techniques and aortic cross-clamping.

Measurement data were reported as the mean and standard deviation or as the median and interquartile range based on the reported studies. The proportion per 100 observations with a 95% confidence interval (95% CI) was calculated for each binary outcome. For late mortality following SPE, the incidence rate with a constant event rate was used to account for different follow-up times of the various studies with the total number of events observed within the treatment group out of the total person-year of the follow-up. 

Meta-regression was used to assess the effect of publication year, sex, systemic thrombolysis, contraindication to systemic thrombolytics, preoperative cardiac arrest, inotrope or vasopressor use, preoperative mechanical ventilation, use of CPB, myocardial protective techniques, use of intraoperative hypothermia and aortic cross-clamping percent on hospital mortality after SPE. Heterogeneity among the included studies was assessed using the Cochran Q statistic and the I^2^ test. For the primary outcome, if heterogeneity was significant (I^2^ > 75%), a leave-one-out sensitivity analysis was performed. The publication bias was assessed using a funnel plot and Egger’s regression test. We used a random effect model (inverse variance method) for the entire analysis. The hypothesis testing for equivalence was set at a two-tailed value of 0.05. Analyses were performed using R (version 4.3.3 R Project for Statistical Computing), using the following statistical packages: “meta” and “metafor” within RStudio (2023.12.1+402 “Ocean Storm” Release for windows; Postit: Boston, MA, USA).

## 3. Results

Among the 1949 searched studies in our systematic literature search, 78 studies met our inclusion criteria including 6859 cases that underwent an SPE intervention [6,7,8,9,10,11,12,13,14,15,16,17,18,19,20,21,22,23,24,25,26,27,28,29,30,31,32,33,34,35,36,37,38,39,40,41,42,43,44,45,46,47,48,49,50,51,52,53,54,55,56,57,58,59,60,61,62,63,64,65,66,67,68,69,70,71,72,73,74,75,76,77,78,79,80,81,82,83]. A PRISMA flowchart is shown in Supplementary Figure S1.

The criteria of all included studies are presented in Table 1. The mean age of included patients ranged from 42 to 65 years. The percentage of males ranged from 25.6% to 86.7%. Preoperative cardiac arrest was reported in 57 studies and ranged from 0% to 87.2% of operations with a median preoperative cardiac arrest of 27.6%. The percentage of contraindications to preoperative systemic thrombolysis was reported by 29 studies with a median percent of 30.4% (interquartile range 20.00–45.50) in these studies. The preoperative systemic thrombolysis percent was reported by 35 studies with a median percent of 11.5% (interquartile range 3.65–25.30) in these studies. The use of CPB appeared to be nearly universal (median 100% (IQR: 100–100)). The criteria of the included studies are shown in Table 1.

A quality assessment of all studies was conducted according to the Newcastle–Ottawa scale (NOS) criteria, as shown in Supplementary Table S2. 

### 3.1. Efficacy Outcomes

**Figure 1:**
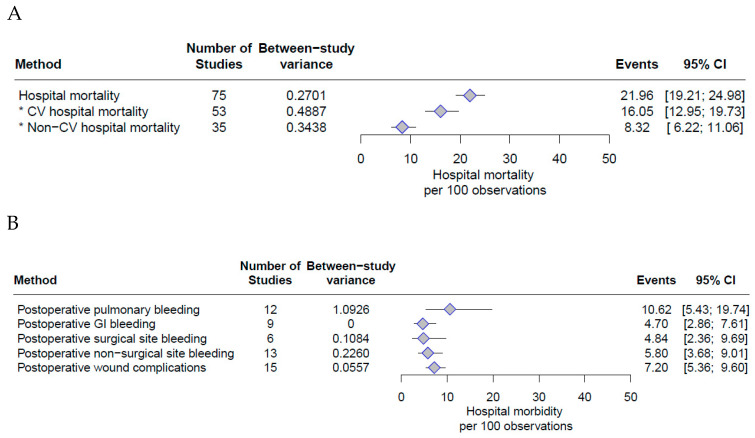
Forest plot of (**A**) hospital mortality (The * refers to the different subgroups of hospital mortality) and (**B**) hospital morbidity.

Point estimates for hospital and late mortality outcomes are reported in Figure 1A and Supplementary Figure S7. Hospital mortality was reported by all 75 studies involving 6779 cases. The hospital mortality was estimated to be 21.96% (95% CI: 19.21–24.98) (Figure 1A). The CV hospital mortality was reported in 53 studies and was estimated to be 16.05% (95% CI: 12.95–19.73). The non-CV hospital mortality was reported in 35 studies and was estimated to be 8.32% (95% CI: 6.22–11.06).

### 3.2. Late All-Cause Mortality

With a weighted median follow-up of 3.05 years, the late CV and non-CV mortality incidence rates were 0.39 per person-year (95% CI: 0.14–0.65) and 0.90 per person-year (95% CI: 0.40–2.06), respectively. (Supplementary Figure S7).

### 3.3. Safety Outcomes

Point estimates for pulmonary bleeding, gastrointestinal bleeding, surgical site bleeding, non-surgical site bleeding and wound complications are reported in Figure 1B.

Pulmonary bleeding was reported by 12 studies, and the incidence was estimated to be 10.62% (95% CI: 5.43–19.74%). Gastrointestinal bleeding was reported by nine studies, and the incidence was estimated to be 4.70% (95% CI: 2.86–7.61). Surgical site bleeding was reported in six studies with an estimated incidence of 4.84% (95% CI: 3.36–9.69%), while non-surgical site bleeding was reported in 13 studies with an estimated incidence of 5.80% (95% CI: 3.68–9.01%). Wound complications were reported in 15 studies with an estimated incidence of 7.2% (95% CI: 5.36–9.60%), Figure 1B.

There were 15 cases of GI bleeding reported, and most of them were due to abdominal surgical operations. Clarke et al.’s 1986 study reported that 10 patients had abdominal surgery for malignant tumor resection, and 4 of them had GI bleeding. Cases of GI bleeding and cerebral strokes were contraindicated for thrombolytics and anticoagulants.

### 3.4. Sensitivity and Subgroup Analyses and Meta-Regression

There is high heterogeneity in hospital mortality with an I2 of 73%. To explore such reasons for heterogeneity, we performed a leave-one-out analysis that showed the robustness of the obtained estimate for hospital mortality (Supplementary Figure S4). Additionally, cumulative meta-analysis showed a decline in hospital mortality for SPE from 42.86% in 1965 to 20.56% in 2024. 

**Figure 2:**
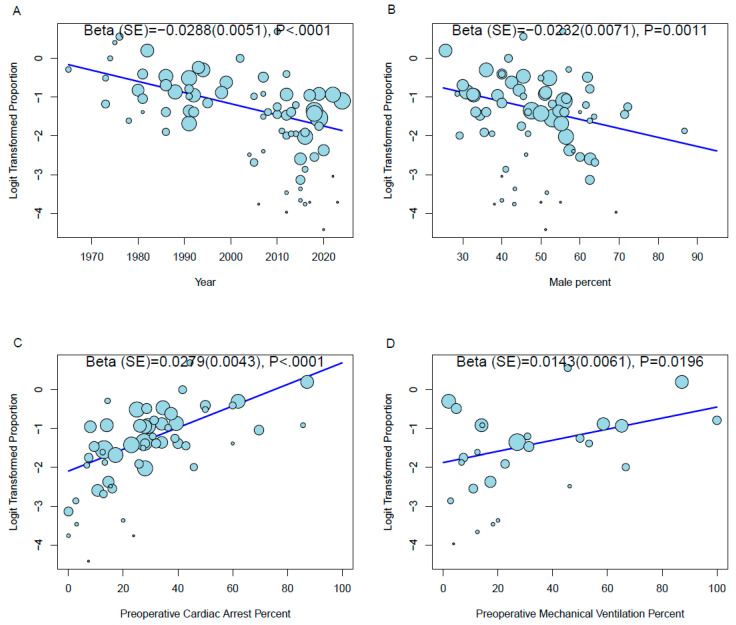
Bubble plots of meta-regression of (**A**) publication year, (**B**) percentage of males, (**C**) preoperative cardiac arrest and (**D**) preoperative mechanical ventilation on hospital mortality outcome.

Meta-regression analyses were performed to evaluate the impact of different variables on hospital mortality and found that the publication year (Figure 2A) (beta = −0.0288 ± 0.0051, *p* < 0.0001) and percentage of males (Figure 2B) (beta = −0.0232 ± 0.0071, *p* = 0.0011) were associated with lower hospital mortality, while preoperative cardiac arrest (Figure 2C) (beta = −0.0288 ± 0.0051, *p* < 0.0001), the need for inotropes or vasopressors (beta = 0.0137 ± 0.0042, *p* = 0.0012) and preoperative mechanical ventilation (Figure 2D) (beta = 0.0143 ± 0.0061, *p* = 0.0196) were associated with higher hospital mortality (Table 2 and Supplementary Figure S3).

There was no observed publication bias either visually by inspecting the symmetry of the funnel plot or statistically by using Egger’s test (estimate = 0.2246 ± 0.2986, *p* = 0.4507), Supplementary Figure S4.

## 4. Discussion

This meta-analysis and systematic review examined the efficacy of SPE, as well as short- and long-term outcomes including CV and non-CV mortality in 78 studies, which included 6859 cases that underwent an SPE. The analysis demonstrated a hospital mortality rate of approximately 22%, with a CV mortality rate of 16%. Additionally, there were long-term CV and non-CV mortality rates of 39 per 100 person-year and 90 deaths per 100 person-year, respectively. The median preoperative cardiac arrest rate was approximately 28%, with the use of CPB universally in patients that underwent SPE.

The in-hospital mortality rates, as well as CV and non-CV mortality rates, reported in this study are similar to the reported mortality rates by Karla et al. who reported an in-hospital mortality rate of 26.3% [4]. A study was conducted by Kilic et al. using a weighted nationwide inpatient sample, which included 1050 participating institutions in 44 states and identified 2709 patients that underwent an SPE for a PE. In this study, they reported an in-hospital mortality rate of 27.2% and identified that the comorbidity index and black race were independently associated with inpatient mortality following SPE [84].

A retrospective study performed by Hartman et al. reported a 30-day mortality rate of 4.2% for all comers but illustrated that patients that were unstable had a higher 30-day mortality rate of 12.5% compared to stable patients who had a 30-day mortality rate of 1.4% [6]. Studies have also shown that mortality rates are higher following cardiac arrest, which could explain the reported in-hospital mortality rate of 27% in this study, given that 28% of patients that underwent SPE had preoperative cardiac arrest. Stein and colleagues reported an operative mortality rate of 59% in patients who had preoperative cardiac arrest compared to a rate of 20% in patients who did not have preoperative cardiac arrest [85].

Furthermore, we found in this study that there is a decline in the in-hospital mortality rate following SPE. It decreased from 42.86% in 1965 to 20.56% in 2024. Studies have previously shown this reduction in mortality over time [86]. This trend is likely due to improvements in the diagnosis of PE, the stabilization of the patient and early intervention. There is also likely a significant selection bias at work, as the dramatic improvement in catheter-based interventions has offered many patients embolectomy in the absence of surgery. This lack of randomization is a major confounder of such a retrospective meta-analysis. This review supports the concept that in appropriately selected patients, surgical embolectomy may be performed safely and with a good outcome; it does not argue against the utility of popular catheter-based techniques/approaches that have rapidly evolved from catheter-directed thrombolysis to ultrasound-augmented thrombolysis and to multiple generations of percutaneous thrombectomy devices.

Among the included studies, there was an apparent trend toward higher inotrope/vasopressor use with RV dysfunction, in studies that reported both variables, but this was statistically insignificant (*p*- for trend = 0.347). The hospital mortality was mainly due to cardiovascular comorbidities which included the need for inotropes or vasopressors, preoperative mechanical ventilation, shock and cardiac arrest. The weighted median follow-up was 3.05 years. Late mortality causes included both CV and non-CV causes. Cardiovascular comorbidities such as hypertension and heart failure and non-CV causes such as malignant neoplasms are the most common causes for late mortality.

Finally, this study has limitations that include the lack of demographic data such as race in the majority of included studies, since previous studies have shown an association between race and in-hospital mortality following SPE. Specifically, the black and African American race was associated with higher mortality rates compared to white Americans [84]. Additionally, hemodynamic information was not present in a reasonable number of the included studies. It would have been interesting to observe if there were differences in hospital mortality following SPE in stable and unstable patients or to understand the baseline presentation of the patient and why that contributed to a hospital mortality rate of approximately 27%; however, we were able to identify some predictors of mortality such as an earlier era of surgery, prior cardiac arrest, need for preoperative mechanical ventilation and the need for vasopressors or inotropes. There is a discernible lack of data on the institution of ECMO among included patients. There is a need to evaluate other late outcomes, such as the rate of development of chronic pulmonary hypertension in patients who undergo SPE for acute PE.

## 5. Conclusions

In conclusion, this meta-analysis and systematic review demonstrates acceptable perioperative mortality rates and late CV and non-CV mortality in patients who undergo SPE for massive or submassive PE. There is a noticeably reduced mortality rate with more recent studies using SPE.

## Figures and Tables

**Table 1:** The criteria of the included studies.

	Country	Number of Patients	Age, Years	Mean Follow-Up (Year)	Male Percent
Hartman 2015 [6]	USA	96	57.70	2.50	62.50
Ahmed 2008 [7]	USA	15	59.60		46.70
Alqahtani 2019 [8]	USA	3486	56.00		53.00
Amirghofran 2007 [9]	Iran	11	45.60	3.00	63.60
Argyriou 2024 [10]	England	256	54.00		55.90
Azari 2015 [11]	Iran	30	56.10	3.50	43.33
Barrett 2010 [12]	UK/Sydney	9	62.00		55.60
Bauer 1991 [13]	Switzerland	44	49.00	4.60	54.50
Bennett 2015 [14]	USA	40	50.33		40.00
Berger 1973 [15]	USA	17			52.90
Biglioli 1991 [16]	Italy	11			
Bottzauw 1981 [17]	USA	23	53.00		56.50
Boulafendis 1991 [18]	USA	16	51.50	5.04	62.50
Cale 2002 [19]	Singapore	12			41.70
Clarke 1986 [20]	England	55			45.50
Dauphine 2005 [21]	USA	11	48.50	0.75	45.50
De Weese1976 [22]	Germany	11	42.30		45.50
DiChiacchio 1986 [23]	USA	90	53.56		50.00
Digonnet 2007 [24]	France	21	62.00	4.75	61.90
Doerge 1998 [25]	Germany	41	51.10	10.58	51.20
Dohle 2018 [26]	Germany	175	59.30	4.60	50.00
Edelman 2016 [27]	Australia	37	57.00	0.12	41.00
Estrer 1981 [28]	USA	5	43.60		60.00
Fedorov 2022 [29]	Russia	10	54.60		40.00
Glassford 1981 [30]	USA	20	57.10		40.00
Gray 1988 [31]	England	71	43.10	7.88	31.00
Greelish 2011 [32]	USA	15	57.00	2.00	86.70
Hajizadeh 2017 [33]	Iran	36	50.80	0.50	38.90
Hennig 1974 [34]	Germany	6		1.67	
Jako1995 [35]	Germany	25	57.00		40.00
Jaumin 1986 [36]	Belgium	23			
Keeling 2016 [37]	USA	214	56.00		56.40
Keeling 2016 [38]	USA	44	51.60	2.52	43.20
Khoury 1992 [39]	Australia	61	53.00		32.80
Kieny 1991 [40]	France	134	55.00		55.20
Konstantinov 2007 [41]	Australia	7	46.40	4.17	28.60
Laas 1993 [42]	Germany	34		4.90	
Leacche 2005 [43]	USA	47	59.00	2.25	63.80
Lehnert 2012 [44]	Denmark	33	55.00	5.20	51.50
Lund 1986 [45]	Denmark	25	52.00	3.90	56.00
Malekan 2012 [46]	USA	26	59.10	0.08	69.20
Marshall 2012 [47]	Australia	10	49.00	3.25	40.00
Mattox 1982 [48]	USA	39	42.00		25.60
Meyer 1991 [49]	France	96	52.00	4.67	52.10
Meyns 1992 [50]	Belgium	30	47.80	7.25	33.30
Minakawa 2018 [51]	Japan	355	62.10		47.60
Mkalaluh 2019 [52]	Germany	49	58.00	0.08	51.00
Neely 2015 [53]	USA	115	59.00	1.08	62.60
Newcom2022 [54]	USA	16	53.00		44.00
Osborne 2014 [55]	USA	15	48.50	0.09	46.70
Panholzer 2022 [56]	Germany	103	58.40		
Park 2019 [57]	Korea	27	47.30	0.08	45.00
Pasrij 2017 [58]	USA	30	55.50	0.50	50.00
Pasrij 2018 [59]	USA	55	53.00	1.00	60.00
QiMin 2020 [60]	China	41	65.00	2.00	51.20
Rathore 2020 [61]	Australia	82	60.00	3.18	57.30
Rivas 1975 [62]	Germany	5			
Sa 2007 [63]	Korea	12	46.00	8.50	58.30
Salehi 2013 [64]	Iran	16	53.00	2.00	37.50
Sareyyupoglu 2010 [65]	USA	18	60.00	1.33	72.20
Satter 1980 [66]	Germany	36			44.40
Saylam 1978 [67]	USA	8	58.50		62.50
Shiomi 2016 [68]	Japan	31	58.30	3.98	35.50
Spagnolo 2006 [69]	Italy	21			38.10
Stalpaert 1986 [70]	Germany	30	44.50		30.00
Stulz 1994 [71]	Switzerland	50	53.40		36.00
Takahashi 2012 [72]	Japan	24	59.90	0.57	29.20
Taniguchi 2012 [73]	Japan	32	57.00	0.08	34.40
Thielmann 2012 [74]	Germany	46	50.50	0.08	32.60
Turnier 1973 [75]	USA	8	56.80		50.00
Ullman 1999 [76]	Germany	40	55.00	3.75	42.50
Vohr2010 [77]	UK	21	55.00	3.17	71.40
Vossschulte 1965 [78]	Germany	7	48.70		57.10
Wu 2013 [79]	Taiwan	25	49.40	1.58	36.00
Yalamanchili 2004 [80]	USA	13	53.70		46.20
Yavuz 2014 [81]	Turkey	13	61.80	2.08	61.50
Zarrabi 2013 [82]	Iran	30			
Zielinski 2023 [83]	Poland	20	53.65	3.83	55.00

**Table 2:** Meta regression of hospital mortality.

Variables	Beta ± SE, *p*-Value
Year	−0.0288 ± 0.0051, *p* < 0.0001
Male Percent	−0.0232 ± 0.0071, *p* = 0.0011
Systemic Thrombolysis Percent	−0.0136 ± 0.0078, *p* = 0.0785
Systemic Thrombolytics Contraindication Percent	0.0075 ± 0.0062, *p* = 0.2261
Preoperative Cardiac Arrest Percent	0.0279 ± 0.0043, *p* < 0.0001
Need for Inotropes or Vasopressors Percent	0.0137 ± 0.0042, *p* = 0.0012
Preoperative Mechanical Ventilation Percent	0.0143 ± 0.0061, *p* = 0.0196
Use Of Cardiopulmonary Bypass Percent	−0.0060 ± 0.0040, *p* = 0.1325
Use Of Myocardial Protective Techniques Percent	−0.0032 ± 0.0050, *p* = 0.5243
Use Of Intraoperative Hypothermia Percent	0.0013 ± 0.0042, *p* = 0.7656
Use Of Aortic Cross-Clamping Percent	−0.0012 ± 0.0036, *p* = 0.7454

Beta (regression coefficient): the negative value reflects inverse association with the hospital mortality outcome.

## Data Availability

The data presented in this study are available on request from the corresponding authors. The data are not publicly available due to institutional policy.

## Supplementary Materials

The following supporting information can be downloaded at https://www.mdpi.com/article/10.3390/jcm13144076/s1. Figure S1: A PRISMA flowchart of the included studies, Figure S2: Forest plot of hospital mortality, Figure S3: Cumulative meta-analysis showing a decrease in hospital mortality for surgical pulmonary embolectomy from 42.86% in 1965 to 20.56% in 2024, Figure S4: Hospital mortality (A) leave-one-out and (B) funnel plot, Figure S5: Forest plot for hospital CV mortality, Figure S6: Forest plot of non-CV hospital mortality, Figure S7: (A) Late cardiovascular (CV) mortality and (B) late non-CV mortality, Figure S8: Forest plot of postoperative pulmonary bleeding, Figure S9: Forest plot of postoperative gastrointestinal (GI) bleeding, Figure S10: Forest plot bleeding at surgical site, Figure S11: Forest plot bleeding at non-surgical site, Figure S12: Wound complications, Table S1: Intraoperative criteria of included studies, Table S2: Newcastle–Ottawa scale for quality assessment of included studies.

## References

[1] Goldhaber S.Z. (2013). Surgical Pulmonary Embolectomy: The Resurrection of an Almost Discarded Operation. Tex. Heart Inst. J..

[2] Konstantinides S.V., Meyer G., Becattini C., Bueno H., Geersing G.-J., Harjola V.-P., Huisman M.V., Humbert M., Jennings C.S., Jiménez D. (2020). 2019 ESC Guidelines for the Diagnosis and Management of Acute Pulmonary Embolism Developed in Collaboration with the European Respiratory Society (ERS). Eur. Heart J..

[3] Duffett L., Castellucci L.A., Forgie M.A. (2020). Pulmonary Embolism: Update on Management and Controversies. BMJ.

[4] Kalra R., Bajaj N.S., Arora P., Arora G., Crosland W.A., McGiffin D.C., Ahmed M.I. (2017). Surgical Embolectomy for Acute Pulmonary Embolism: Systematic Review and Comprehensive Meta-Analyses. Ann. Thorac. Surg..

[5] Wells G.A., Shea B., O’Connell D., Peterson J., Welch V., Losos M., Tugwell P. The Newcastle-Ottawa Scale (NOS) for Assessing the Quality of Nonrandomised Studies in Meta-Analyses. https://web.archive.org/web/20210716121605id_/http://www3.med.unipmn.it/dispense_ebm/2009-2010/Corso%20Perfezionamento%20EBM_Faggiano/NOS_oxford.pdf.

[6] Hartman A.R., Manetta F., Lessen R., Pekmezaris R., Kozikowski A., Jahn L., Akerman M., Lesser M.L., Glassman L.R., Graver M. (2015). Acute Surgical Pulmonary Embolectomy: A 9-Year Retrospective Analysis. Tex. Heart Inst. J..

[7] Ahmed P., Khan A.A., Smith A., Pagala M., Abrol S., Cunningham J.N., Vaynblat M. (2008). Expedient Pulmonary Embolectomy for Acute Pulmonary Embolism: Improved Outcomes. Interact. Cardiovasc. Thorac. Surg..

[8] Alqahtani F., Munir M.B., Aljohani S., Tarabishy A., Almustafa A., Alkhouli M. (2019). Surgical Thrombectomy for Pulmonary Embolism: Updated Performance Rates and Outcomes. Tex. Heart Inst. J..

[9] Amirghofran A.A., Emami Nia A., Javan R. (2007). Surgical Embolectomy in Acute Massive Pulmonary Embolism. Asian Cardiovasc. Thorac. Ann..

[10] Argyriou A., Vohra H., Chan J., Ahmed E.M., Rajakaruna C., Angelini G.D., Fudulu D.P. (2024). Incidence and Outcomes of Surgical Pulmonary Embolectomy in the UK. Br. J. Surg..

[11] Azari A., Bigdelu L., Moravvej Z. (2015). Surgical Embolectomy in the Management of Massive and Sub-Massive Pulmonary Embolism: The Results of 30 Consecutive Ill Patients. ARYA Atheroscler..

[12] Barrett N.A., Byrne A., Delaney A., Hibbert M., Ramakrishnan N. (2010). Management of Massive Pulmonary Embolism: A Retrospective Single-Centre Cohort Study. Crit. Care Resusc..

[13] Bauer E., Laske A., Segesser L., Carrel T., Turina M. (1991). Early and Late Results after Surgery for Massive Pulmonary Embolism. Thorac. Cardiovasc. Surg..

[14] Bennett J.M., Pretorius M., Ahmad R.M., Eagle S.S. (2015). Hemodynamic Instability in Patients Undergoing Pulmonary Embolectomy: Institutional Experience. J. Clin. Anesth..

[15] Berger R.L. (1973). Pulmonary Embolectomy with Preoperative Circulatory Support. Ann. Thorac. Surg..

[16] Biglioli P., Alamanni F., Spirito R., Arena V. (1991). From deep venous thrombosis to pulmonary embolism. Cardiologia.

[17] Bøttzauw J., Vejlsted H., Albrechtsen O. (1981). Pulmonary Embolectomy Using Extracorporeal Circulation. Thorac. Cardiovasc. Surg..

[18] Boulafendis D., Bastounis E., Panayiotopoulos Y.P., Papalambros E.L. (1991). Pulmonary Embolectomy (Answered and Unanswered Questions). Int. Angiol. J. Int. Union Angiol..

[19] Caleb M.G. (2002). Massive Pulmonary Embolism with Haemodynamic Collapse. Singap. Med. J..

[20] Clarke D.B., Abrams L.D. (1986). Pulmonary Embolectomy: A 25 Year Experience. J. Thorac. Cardiovasc. Surg..

[21] Dauphine C., Omari B. (2005). Pulmonary Embolectomy for Acute Massive Pulmonary Embolism. Ann. Thorac. Surg..

[22] De Weese J.A. (1976). The Role of Pulmonary Embolectomy in Venous Thromboembolism. J. Cardiovasc. Surg..

[23] DiChiacchio L., Pasrija C., Boulos F.M., Ramani G., Jeudy J., Deatrick K.B., Griffith B.P., Kon Z.N. (2019). Occult Chronic Thromboembolic Disease in Patients Presenting for Surgical Pulmonary Embolectomy. Ann. Thorac. Surg..

[24] Digonnet A., Moya-Plana A., Aubert S., Flecher E., Bonnet N., Leprince P., Pavie A., Gandjbakhch I. (2007). Acute Pulmonary Embolism: A Current Surgical Approach. Interdiscip. CardioVascular Thorac. Surg..

[25] Doerge H., Schoendube F., Voß M., Seipelt R., Messmer B. (1999). Surgical Therapy of Fulminant Pulmonary Embolism: Early and Late Results. Thorac. Cardiovasc. Surg..

[26] Dohle K., Dohle D.-S., El Beyrouti H., Buschmann K., Emrich A.L., Brendel L., Vahl C.-F. (2018). Short- and Long-Term Outcomes for the Surgical Treatment of Acute Pulmonary Embolism. Innov. Surg. Sci..

[27] Edelman J.J., Okiwelu N., Anvardeen K., Joshi P., Murphy B., Sanders L.H., Newman M.A., Passage J. (2016). Surgical Pulmonary Embolectomy: Experience in a Series of 37 Consecutive Cases. Heart Lung Circ..

[28] Estrera A.S., Platt M.R., Mills L.J. (1981). Pulmonary Embolectomy for Massive Pulmonary Embolism. Tex. Med..

[29] Fedorov S.A., Pichugin V.V., Chiginev V.A., Brichkin Y.D., Kulkarni S.V., Tselousova L.M., Kalinina M.L., Taranov E.V., Domnin S.E. (2022). Domnin Possibilities and Perspectives of Retrograde Pulmonary Artery Perfusion as a Component of Surgical Treatment of Pulmonary Embolism. Opera Med. Physiol..

[30] Glassford D.M., Alford W.C., Burrus G.R., Stoney W.S., Thomas C.S. (1981). Pulmonary Embolectomy. Ann. Thorac. Surg..

[31] Gray H.H., Morgan J.M., Paneth M., Miller G.A. (1988). Pulmonary Embolectomy for Acute Massive Pulmonary Embolism: An Analysis of 71 Cases. Heart.

[32] Greelish J.P., Leacche M., Solenkova N.S., Ahmad R.M., Byrne J.G. (2011). Improved Midterm Outcomes for Type A (Central) Pulmonary Emboli Treated Surgically. J. Thorac. Cardiovasc. Surg..

[33] Hajizadeh R., Ghaffari S., Habibzadeh A., Safaei N., Mohammadi K., Ranjbar A., Ghodratizadeh S. (2017). Outcome of Surgical Embolectomy in Patients with Massive Pulmonary Embolism with and without Cardiopulmonary Resuscitation. Pol. J. Thorac. Cardiovasc. Surg..

[34] Hennig K., Franke D., Fenn K. (1974). Pulmonary embolectomy (report on 3 personal cases). VASA Z. Gefasskrankh..

[35] Jakob H., Vahl C., Lange R., Micek M., Tanzeem A., Hagl S. (1995). Modified Surgical Concept for Fulminant Pulmonary Embolism. Eur. J. Cardio-Thorac. Surg..

[36] Jaumin P., Moriau M., el Gariani A., Rubay J., Baele P., Dautrebande J., Goenen M., Servaye-Kestens Y., Ponlot R. (1986). Pulmonary embolectomy. Clinical experience. Acta Chir. Belg..

[37] Keeling W.B., Sundt T., Leacche M., Okita Y., Binongo J., Lasajanak Y., Aklog L., Lattouf O.M. (2016). Outcomes After Surgical Pulmonary Embolectomy for Acute Pulmonary Embolus: A Multi-Institutional Study. Ann. Thorac. Surg..

[38] Keeling W.B., Leshnower B.G., Lasajanak Y., Binongo J., Guyton R.A., Halkos M.E., Thourani V.H., Lattouf O.M. (2016). Midterm Benefits of Surgical Pulmonary Embolectomy for Acute Pulmonary Embolus on Right Ventricular Function. J. Thorac. Cardiovasc. Surg..

[39] Khoury E., Rabinov M., Davis B.B., Stirling G.R. (1992). Pulmonary Embolectomy in the Management of Massive Pulmonary Embolism. Australas. J. Card. Thorac. Surg..

[40] Kieny R., Charpentier A., Kieny M.T. (1991). What Is the Place of Pulmonary Embolectomy Today?. J. Cardiovasc. Surg..

[41] Konstantinov I.E., Saxena P., Koniuszko M.D., Alvarez J., Newman M.A.J. (2007). Acute Massive Pulmonary Embolism with Cardiopulmonary Resuscitation: Management and Results. Tex. Heart Inst. J..

[42] Laas J., Schmid C., Albes J.M., Borst H.G. (1993). Surgical aspects of fulminant pulmonary embolism. Z. Kardiol..

[43] Leacche M., Unic D., Goldhaber S.Z., Rawn J.D., Aranki S.F., Couper G.S., Mihaljevic T., Rizzo R.J., Cohn L.H., Aklog L. (2005). Modern Surgical Treatment of Massive Pulmonary Embolism: Results in 47 Consecutive Patients after Rapid Diagnosis and Aggressive Surgical Approach. J. Thorac. Cardiovasc. Surg..

[44] Lehnert P., Møller C.H., Carlsen J., Grande P., Steinbrüchel D.A. (2012). Surgical Treatment of Acute Pulmonary Embolism—A 12-Year Retrospective Analysis. Scand. Cardiovasc. J..

[45] Lund O., Nielsen T., Schifter S., Roenne K. (1986). Treatment of Pulmonary Embolism with Full-Dose Heparin, Streptokinase or Embolectomy—Results and Indications. Thorac. Cardiovasc. Surg..

[46] Malekan R., Saunders P.C., Yu C.J., Brown K.A., Gass A.L., Spielvogel D., Lansman S.L. (2012). Peripheral Extracorporeal Membrane Oxygenation: Comprehensive Therapy for High-Risk Massive Pulmonary Embolism. Ann. Thorac. Surg..

[47] Marshall L., Mundy J., Garrahy P., Christopher S., Wood A., Griffin R., Shah P. (2012). Surgical Pulmonary Embolectomy: Mid-term Outcomes. ANZ J. Surg..

[48] Mattox K.L., Feldtman R.W., Beall A.C., DeBAKEY M.E. (1982). Pulmonary Embolectomy for Acute Massive Pulmonary Embolism. Ann. Surg..

[49] Meyer G., Tamisier D., Sors H., Stern M., Vouhé P., Makowski S., Neveux J.-Y., Leca F., Even P. (1991). Pulmonary Embolectomy: A 20-Year Experience at One Center. Ann. Thorac. Surg..

[50] Meyns B., Sergeant P., Flameng W., Daenen W. (1992). Surgery for Massive Pulmonary Embolism. Acta Cardiol..

[51] Minakawa M., Fukuda I., Miyata H., Motomura N., Takamoto S., Taniguchi S., Daitoku K., Kondo N., Japan Cardiovascular Surgery Database Organization (2018). Outcomes of Pulmonary Embolectomy for Acute Pulmonary Embolism. Circ. J..

[52] Mkalaluh S., Szczechowicz M., Karck M., Szabo G. (2019). Twenty-Year Results of Surgical Pulmonary Thromboembolectomy in Acute Pulmonary Embolism. Scand. Cardiovasc. J..

[53] Neely R.C., Byrne J.G., Gosev I., Cohn L.H., Javed Q., Rawn J.D., Goldhaber S.Z., Piazza G., Aranki S.F., Shekar P.S. (2015). Surgical Embolectomy for Acute Massive and Submassive Pulmonary Embolism in a Series of 115 Patients. Ann. Thorac. Surg..

[54] Newcomb G., Wilson B.L., White R.J., Goldman B., Lachant N.A., Lachant D.J. (2022). An Untapped Resource: Characteristics of Thrombus Recovered from Intermediate or High Risk Pulmonary Embolus Patients. Cardiovasc. Pathol..

[55] Osborne Z.J., Rossi P., Aucar J., Dharamsy S., Cook S., Wheatley B. (2014). Surgical Pulmonary Embolectomy in a Community Hospital. Am. J. Surg..

[56] Panholzer B., Gravert H., Borzikowsky C., Huenges K., Schoettler J., Schoeneich F., Attmann T., Haneya A., Frank D., Cremer J. (2022). Outcome after Surgical Embolectomy for Acute Pulmonary Embolism. J. Cardiovasc. Med..

[57] Park J., Lim S.-H., Hong Y.S., Park S., Lee C.J., Lee S.O. (2019). Acute Pulmonary Thromboembolism: 14 Years of Surgical Experience. Korean J. Thorac. Cardiovasc. Surg..

[58] Pasrija C., Shah A., Sultanik E., Rouse M., Ghoreishi M., Bittle G.J., Boulos F., Griffith B.P., Kon Z.N. (2017). Minimally Invasive Surgical Pulmonary Embolectomy: A Potential Alternative to Conventional Sternotomy. Innov. Technol. Tech. Cardiothorac. Vasc. Surg..

[59] Pasrija C., Kronfli A., Rouse M., Raithel M., Bittle G.J., Pousatis S., Ghoreishi M., Gammie J.S., Griffith B.P., Sanchez P.G. (2018). Outcomes after Surgical Pulmonary Embolectomy for Acute Submassive and Massive Pulmonary Embolism: A Single-Center Experience. J. Thorac. Cardiovasc. Surg..

[60] Wang Q., Chen L., Chen D., Qiu H., Huang Z., Dai X., Huang X., Lin F., Chen H. (2020). Clinical Outcomes of Acute Pulmonary Embolectomy as the First-Line Treatment for Massive and Submassive Pulmonary Embolism: A Single-Centre Study in China. J. Cardiothorac. Surg..

[61] Rathore K.S., Weightman W., Passage J., Joshi P., Sanders L., Newman M. (2020). Risk Stratification Using Serum Lactate in Patients Undergoing Surgical Pulmonary Embolectomy. J. Cardiothorac. Surg..

[62] Rivas J., Bircks W., Nier H., Schneider E., Tarbiat S. (1975). Lungenembolie. DMW—Dtsch. Med. Wochenschr..

[63] Sa Y., Choi S., Lee J., Kwon J., Moon S., Jo K., Wang Y., Kim S., Kim P., Jung H. (2007). Off-Pump Open Pulmonary Embolectomy for Patients with Major Pulmonary Embolism. Heart Surg. Forum.

[64] Salehi R., Ansarin K. (2013). Surgical Embolectomy in Treating Acute Massive Pulmonary Embolism. J. Pak. Med. Assoc..

[65] Sareyyupoglu B., Greason K.L., Suri R.M., Keegan M.T., Dearani J.A., Sundt T.M. (2010). A More Aggressive Approach to Emergency Embolectomy for Acute Pulmonary Embolism. Mayo Clin. Proc..

[66] Satter P. (1980). Pulmonary Embolectomy. Indication and Results. Ann. Radiol..

[67] Saylam A., Melo J.Q., Ahmad A., Chapman R.D., Wood J.A., Starr A. (1978). Pulmonary Embolectomy. West. J. Med..

[68] Shiomi D., Kiyama H., Shimizu M., Yamada M., Shimada N., Takahashi A., Kaki N. (2017). Surgical Embolectomy for High-Risk Acute Pulmonary Embolism Is Standard Therapy. Interdiscip. CardioVascular Thorac. Surg..

[69] Spagnolo S., Grasso M.A., Tesler U.F. (2006). Retrograde Pulmonary Perfusion Improves Results in Pulmonary Embolectomy for Massive Pulmonary Embolism. Tex. Heart Inst. J..

[70] Stalpaert G., Suy R., Daenen W., Flameng W., Sergeant P., Nevelsteen A., Lauwers P., De Geest H., Van Elst F. (1986). Surgical Treatment of Acute, Massive Lung Embolism. Results and Follow-Up. Acta Chir. Belg..

[71] Stulz P., Schlapfer R., Feer R., Habicht J., Gradel E. (1994). Decision Making in the Surgical Treatment of Massive Pulmonary Embolism. Eur. J. Cardio-Thorac. Surg..

[72] Takahashi H., Okada K., Matsumori M., Kano H., Kitagawa A., Okita Y. (2012). Aggressive Surgical Treatment of Acute Pulmonary Embolism with Circulatory Collapse. Ann. Thorac. Surg..

[73] Taniguchi S., Fukuda W., Fukuda I., Watanabe K.-I., Saito Y., Nakamura M., Sakuma M. (2012). Outcome of Pulmonary Embolectomy for Acute Pulmonary Thromboembolism: Analysis of 32 Patients from a Multicentre Registry in Japan. Interact. Cardiovasc. Thorac. Surg..

[74] Thielmann M., Pasa S., Wendt D., Price V., Marggraf G., Neuhauser M., Piotrowski A., Jakob H. (2012). Prognostic Significance of Cardiac Troponin I on Admission for Surgical Treatment of Acute Pulmonary Embolism: A Single-Centre Experience over More than 10 Years. Eur. J. Cardiothorac. Surg..

[75] Turnier E., Hill J.D., Kerth W.J., Gerbode F. (1973). Massive Pulmonary Embolism. Am. J. Surg..

[76] Ullmann M., Hemmer W., Hannekum A. (1999). The Urgent Pulmonary Embolectomy: Mechanical Resuscitation in the Operating Theatre Determines the Outcome. Thorac. Cardiovasc. Surg..

[77] Vohra H.A., Whistance R.N., Mattam K., Kaarne M., Haw M.P., Barlow C.W., Tsang G.M.K., Livesey S.A., Ohri S.K. (2010). Early and Late Clinical Outcomes of Pulmonary Embolectomy for Acute Massive Pulmonary Embolism. Ann. Thorac. Surg..

[78] Vossschulte K., Stiller H., Eisenreich F. (1965). Emergency Embolectomy by the Transsternal Approach in Acute Pulmonary Embolism. Surgery.

[79] Wu M.-Y., Liu Y.-C., Tseng Y.-H., Chang Y.-S., Lin P.-J., Wu T.-I. (2013). Pulmonary Embolectomy in High-Risk Acute Pulmonary Embolism: The Effectiveness of a Comprehensive Therapeutic Algorithm Including Extracorporeal Life Support. Resuscitation.

[80] Yalamanchili K., Fleisher A.G., Lehrman S.G., Axelrod H.I., Lafaro R.J., Sarabu M.R., Zias E.A., Moggio R.A. (2004). Open Pulmonary Embolectomy for Treatment of Major Pulmonary Embolism. Ann. Thorac. Surg..

[81] Yavuz S., Toktas F., Goncu T., Eris C., Gucu A., Ay D., Erdolu B., Tenekecioglu E., Karaagac K., Vural H. (2014). Surgical Embolectomy for Acute Massive Pulmonary Embolism. Int. J. Clin. Exp. Med..

[82] Zarrabi K., Zolghadrasli A., Ostovan M.A., Azimifar A., Malekmakan L. (2013). Residual Pulmonary Hypertension after Retrograde Pulmonary Embolectomy: Long-Term Follow-up of 30 Patients with Massive and Submassive Pulmonary Embolism. Interact. Cardiovasc. Thorac. Surg..

[83] Zieliński D., Zygier M., Dyk W., Wojdyga R., Wróbel K., Pirsztuk E., Szostakiewicz K., Szatkowski P., Darocha S., Kurzyna M. (2023). Acute Pulmonary Embolism with Coexisting Right Heart Thrombi in Transit-Surgical Treatment of 20 Consecutive Patients. Eur. J. Cardio-Thorac. Surg. Off. J. Eur. Assoc. Cardio-Thorac. Surg..

[84] Kilic A., Shah A.S., Conte J.V., Yuh D.D. (2013). Nationwide Outcomes of Surgical Embolectomy for Acute Pulmonary Embolism. J. Thorac. Cardiovasc. Surg..

[85] Stein P.D., Alnas M., Beemath A., Patel N.R. (2007). Outcome of Pulmonary Embolectomy. Am. J. Cardiol..

[86] Poterucha T.J., Bergmark B., Aranki S., Kaneko T., Piazza G. (2015). Surgical Pulmonary Embolectomy. Circulation.

