# Supplementary material for: The Outcomes of Surgical Pulmonary Embolectomy for Pulmonary Embolism: A Meta-Analysis
Source: J Clin Med. 2024 Jul 12;13(14):4076. doi: 10.3390/jcm13144076 (PMC11278425; doi:10.3390/jcm13144076)
Supplement: Supplementary file 1 [file jcm-13-04076-s001.zip › jcm-3073298-supplementary.pdf]

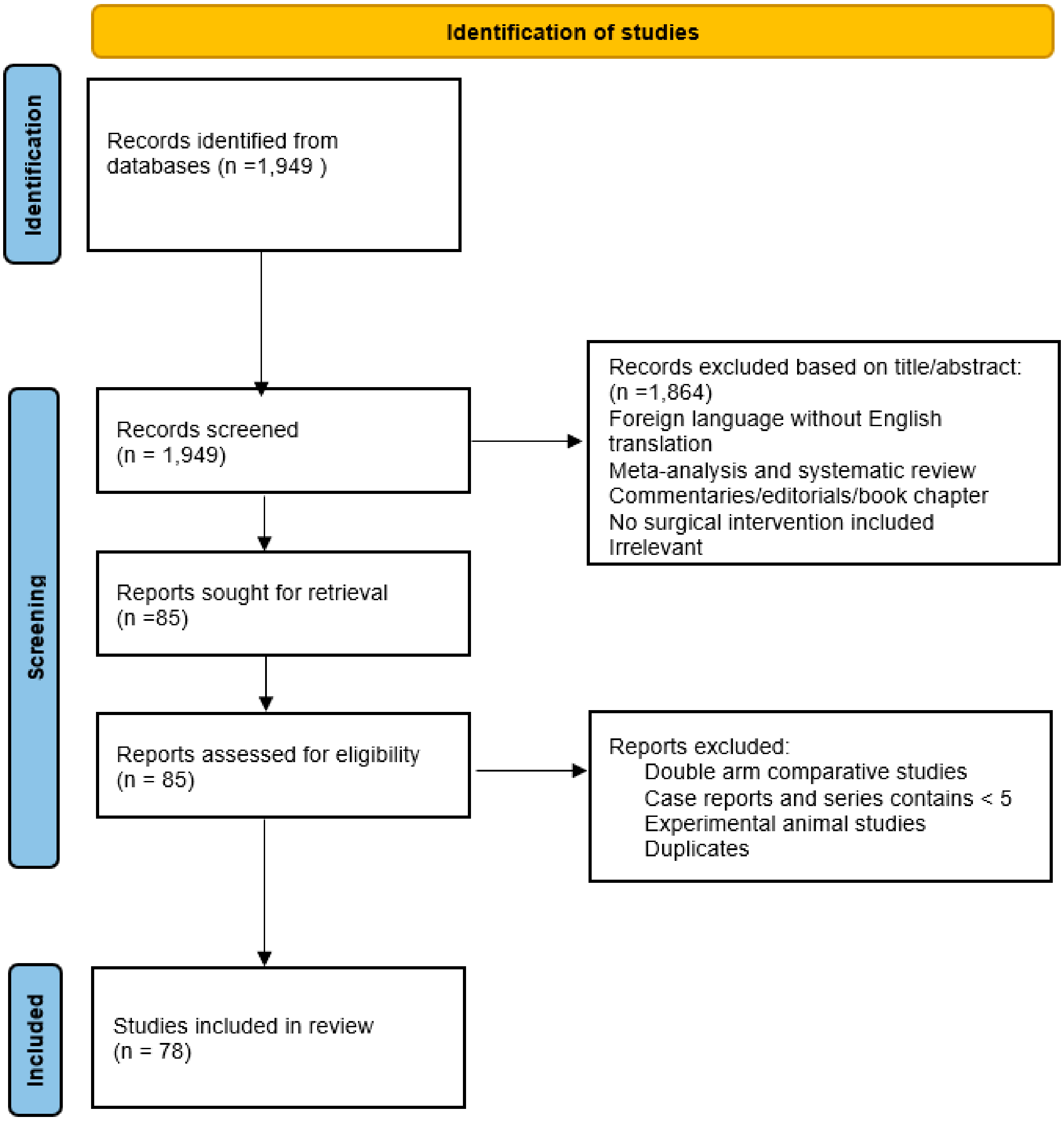

Figure S1. PRISMA flowchart of the included studies.

Study	Events	Total	Weight (common)	Weight (random)	Events per 100 observations IV, Fixed + Random, 95% CI
Barrett 2010	6	9	0.2%	1.0%	66.67 [29.93; 92.51]
De Weese1976	7	11	0.3%	1.1%	63.64 [30.79; 89.07]
Rivas 1975	3	5	0.1%	0.7%	60.00 [14.66; 94.73]
Mattox 1982	22	40	1.0%	2.0%	55.00 [38.49; 70.74]
Hennig 1974	3	6	0.2%	0.8%	50.00 [11.81; 88.19]
Cale2002	6	12	0.3%	1.2%	50.00 [21.09; 78.91]
Laas 1993	15	34	0.8%	1.9%	44.12 [27.19; 62.11]
Vossschulte 1965	3	7	0.2%	0.9%	42.86 [9.90; 81.59]
Stulz 1994	23	54	1.3%	2.1%	42.59 [29.23; 56.79]
Glassford 1981	8	20	0.5%	1.5%	40.00 [19.12; 63.95]
Marshall 2012	4	10	0.2%	1.1%	40.00 [12.16; 73.76]
Clarke 1986	22	57	1.4%	2.1%	38.60 [26.00; 52.43]
Digonnet 2007	8	21	0.5%	1.6%	38.10 [18.11; 61.56]
Turnier 1973	3	8	0.2%	0.9%	37.50 [8.52; 75.51]
Meyer 1991	36	96	2.3%	2.3%	37.50 [27.82; 47.97]
Ullman 1999	14	40	0.9%	1.9%	35.00 [20.63; 51.68]
Stalpaert 1986	10	30	0.7%	1.8%	33.33 [17.29; 52.81]
Boulafendis 1991	5	16	0.3%	1.3%	31.25 [11.02; 58.66]
Satter 1980	11	36	0.8%	1.8%	30.56 [16.35; 48.11]
Gray 1988	21	71	1.5%	2.2%	29.58 [19.33; 41.59]
Doerge 1998	12	41	0.9%	1.9%	29.27 [16.13; 45.54]
Konstantinov 2007	2	7	0.1%	0.8%	28.57 [3.67; 70.96]
Mkalaluh 2019	14	49	1.0%	2.0%	28.57 [16.58; 43.26]
Thielmann 2012	13	46	0.9%	2.0%	28.26 [15.99; 43.46]
Panholzer 2022	29	103	2.1%	2.3%	28.16 [19.73; 37.87]
Khoury 1992	17	61	1.2%	2.1%	27.87 [17.15; 40.83]
Hajizadeh 2017	10	36	0.7%	1.8%	27.78 [14.20; 45.19]
Biglioli 1991	3	11	0.2%	1.0%	27.27 [6.02; 60.97]
Dauphine 2005	3	11	0.2%	1.0%	27.27 [6.02; 60.97]
Bottzauw 1981	6	23	0.4%	1.5%	26.09 [10.23; 48.41]
Argyriou 2024	64	256	4.8%	2.5%	25.00 [19.82; 30.77]
Jako1995	6	25	0.5%	1.5%	24.00 [9.36; 45.13]
Berger 1973	4	17	0.3%	1.2%	23.53 [6.81; 49.90]
Yavuz 2014	3	13	0.2%	1.0%	23.08 [5.04; 53.81]
Sareyyupoglu 2010	4	18	0.3%	1.2%	22.22 [6.41; 47.64]
Minakawa 2018	73	355	5.8%	2.6%	20.56 [16.48; 25.15]
Bauer 1991	9	44	0.7%	1.8%	20.45 [9.80; 35.30]
Estrer 1981	1	5	0.1%	0.5%	20.00 [0.51; 71.64]
Lund 1986	5	25	0.4%	1.4%	20.00 [6.83; 40.70]
Meyns 1992	6	30	0.5%	1.5%	20.00 [7.71; 38.57]
Ahmed 2008	3	15	0.2%	1.1%	20.00 [4.33; 48.09]
Wu 2013	5	25	0.4%	1.4%	20.00 [6.83; 40.70]
Dohle 2018	34	175	2.8%	2.4%	19.43 [13.85; 26.08]
Vohr2010	4	21	0.3%	1.3%	19.05 [5.45; 41.91]
Taniguchi 2012	6	32	0.5%	1.6%	18.75 [7.21; 36.44]
Amirghofran 2007	2	11	0.2%	0.8%	18.18 [2.28; 51.78]
Alqahtani 2019	614	3486	51.0%	2.7%	17.61 [16.36; 18.92]
Saylam 1978	2	12	0.2%	0.8%	16.67 [2.09; 48.41]
Kieny 1991	21	134	1.8%	2.3%	15.67 [9.97; 22.95]
Park 2019	4	27	0.3%	1.3%	14.81 [4.19; 33.73]
Greelish 2011	2	15	0.2%	0.9%	13.33 [1.66; 40.46]
Jaumin 1986	3	23	0.3%	1.1%	13.04 [2.78; 33.59]
Shiomi 2016	4	31	0.4%	1.3%	12.90 [3.63; 29.83]
Salehi 2013	2	16	0.2%	0.9%	12.50 [1.55; 38.35]
Osborne 2014	2	16	0.2%	0.9%	12.50 [1.55; 38.35]
Takahashi 2012	3	25	0.3%	1.1%	12.00 [2.55; 31.22]
Keeling 2016	25	214	2.2%	2.3%	11.68 [7.70; 16.76]
Rathore 2020	7	82	0.6%	1.7%	8.54 [3.50; 16.80]
Sa 2007	1	12	0.1%	0.5%	8.33 [0.21; 38.48]
Yalamanchili 2004	1	13	0.1%	0.5%	7.69 [0.19; 36.03]
Pasrij 2018	4	55	0.4%	1.4%	7.27 [2.02; 17.59]
Neely 2015	8	115	0.8%	1.8%	6.96 [3.05; 13.25]
Leacche 2005	3	47	0.3%	1.2%	6.38 [1.34; 17.54]
Edelman 2016	2	37	0.2%	0.9%	5.41 [0.66; 18.19]
F..dorov 2022	0	10	0.0%	0.3%	0.00 [0.00; 30.85]
Hartman 2015	4	96	0.4%	1.4%	4.17 [1.15; 10.33]
Azari 2015	1	30	0.1%	0.6%	3.33 [0.08; 17.22]
Lehnert 2012	1	33	0.1%	0.6%	3.03 [0.08; 15.76]
Bennett 2015	1	40	0.1%	0.6%	2.50 [0.06; 13.16]
Pasrij 2017	0	20	0.0%	0.3%	0.00 [0.00; 16.84]
Zielinski 2023	0	20	0.0%	0.3%	0.00 [0.00; 16.84]
Spagnolo 2006	0	21	0.0%	0.3%	0.00 [0.00; 16.11]
Keeling 2016	1	44	0.1%	0.6%	2.27 [0.06; 12.02]
Malekan 2012	0	26	0.0%	0.3%	0.00 [0.00; 13.23]
QiMin 2020	0	41	0.0%	0.3%	0.00 [0.00; 8.60]
**Total (common effect, 95% CI)**		**6779**	**100.0%**	.	**20.39 [19.40; 21.42]**
**Total (random effect, 95% CI)**			.	**100.0%**	**21.96 [19.21; 24.98]**

Heterogeneity: $Tau^2 = 0.2701$; $Chi^2 = 269.57$, df = 74 ($P < 0.01$); $I^2 = 73\%$

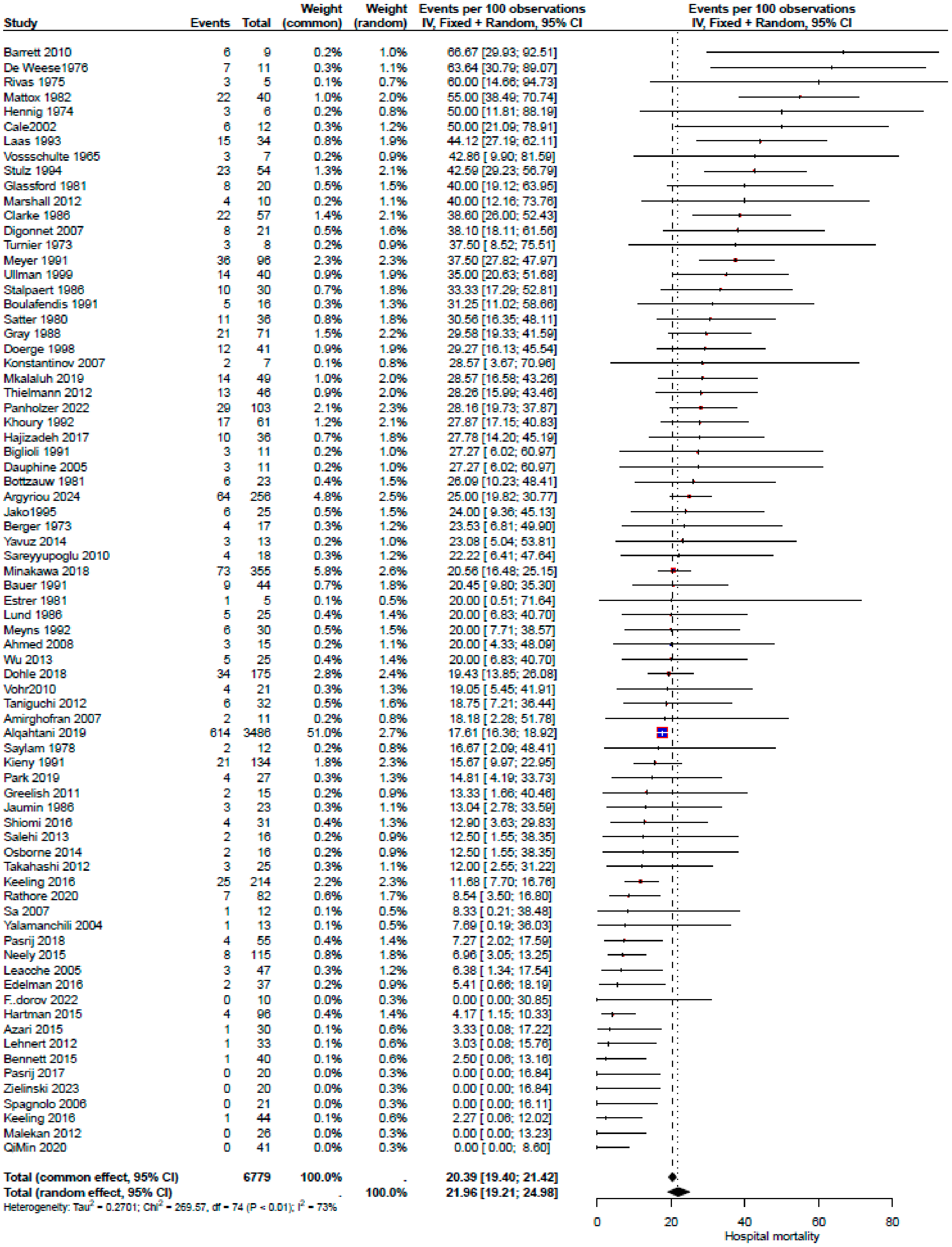

Figure S2: Forest plot of hospital mortality.

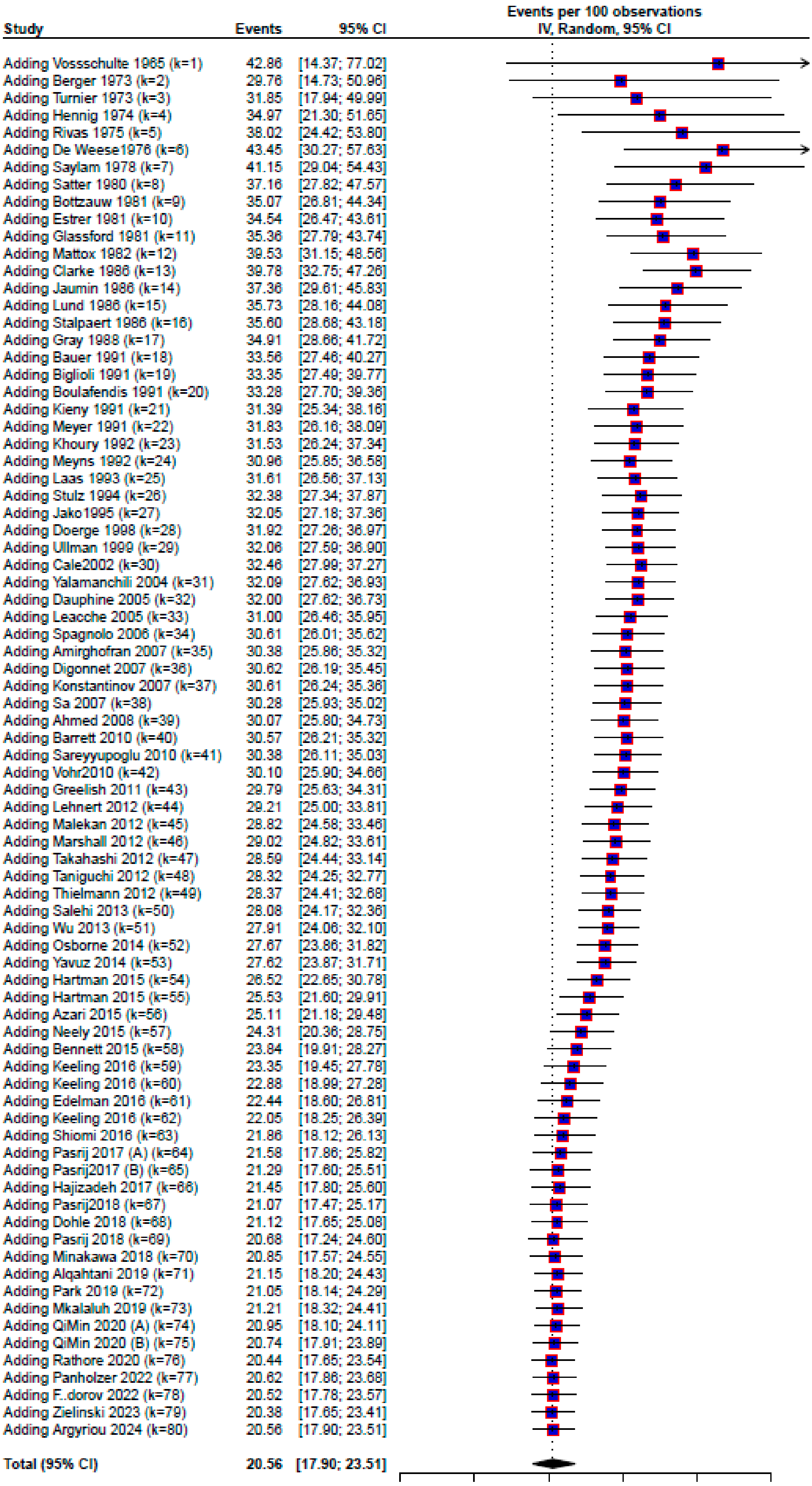

Figure 3. Cumulative meta-analysis showing a decrease in hospital mortality for surgical pulmonary embolectomy from 42.86% in 1965 to 20.56% in 2024.

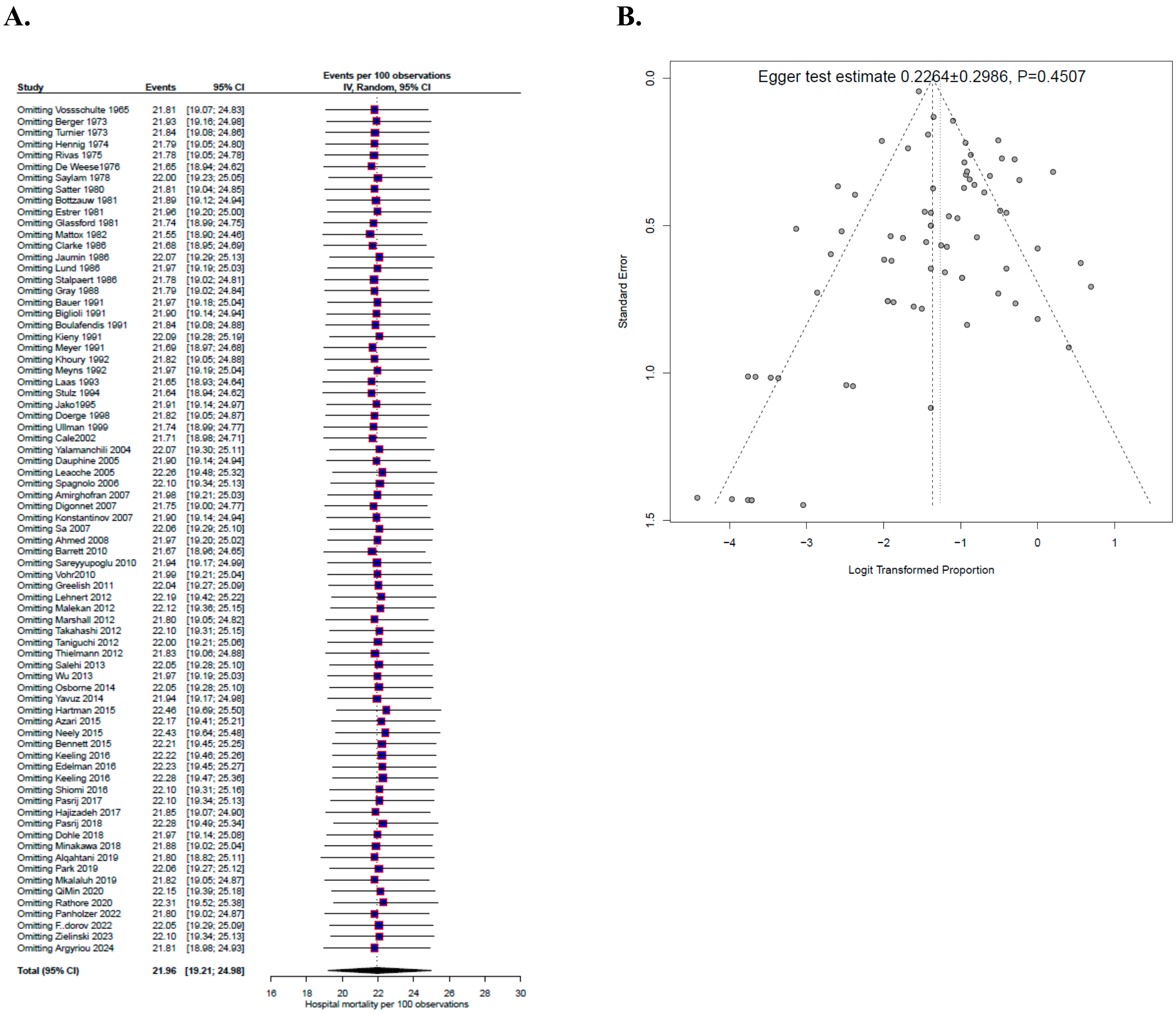

Figure S4. Hospital mortality A) leave-one-out and B) funnel plot.

Study	Events	Total	Weight (common)	Weight (random)	Events per 100 observations IV, Fixed + Random, 95% CI
De Weese1976	7	11	0.9%	1.9%	63.64 [30.79; 89.07]
Mattox 1982	18	40	3.7%	2.8%	45.00 [29.26; 61.51]
Rivas 1975	2	5	0.4%	1.2%	40.00 [5.27; 85.34]
Marshall 2012	4	10	0.9%	1.8%	40.00 [12.16; 73.76]
Laas 1993	13	34	3.0%	2.7%	38.24 [22.17; 56.44]
Turnier 1973	3	8	0.7%	1.6%	37.50 [8.52; 75.51]
Stulz 1994	18	54	4.5%	2.9%	33.33 [21.09; 47.47]
Cale2002	4	12	1.0%	1.9%	33.33 [9.92; 65.11]
Clarke 1986	18	57	4.6%	2.9%	31.58 [19.91; 45.24]
Satter 1980	11	36	2.8%	2.7%	30.56 [16.35; 48.11]
Stalpaert 1986	9	30	2.3%	2.5%	30.00 [14.73; 49.40]
Vossschulte 1965	2	7	0.5%	1.4%	28.57 [3.67; 70.96]
Konstantinov 2007	2	7	0.5%	1.4%	28.57 [3.67; 70.96]
Dauphine 2005	3	11	0.8%	1.7%	27.27 [6.02; 60.97]
Meyer 1991	26	96	7.1%	3.0%	27.08 [18.52; 37.11]
Gray 1988	18	71	5.0%	2.9%	25.35 [15.77; 37.08]
Khoury 1992	15	61	4.2%	2.8%	24.59 [14.46; 37.29]
Thielmann 2012	11	46	3.1%	2.7%	23.91 [12.59; 38.77]
Berger 1973	4	17	1.1%	2.0%	23.53 [6.81; 49.90]
Panholzer 2022	24	103	6.9%	3.0%	23.30 [15.54; 32.66]
Estrer 1981	1	5	0.3%	0.9%	20.00 [0.51; 71.64]
Saylam 1978	2	12	0.6%	1.5%	16.67 [2.09; 48.41]
Sareyyupoglu 2010	3	18	0.9%	1.8%	16.67 [3.58; 41.42]
Hajizadeh 2017	6	36	1.9%	2.4%	16.67 [6.37; 32.81]
Yavuz 2014	2	13	0.6%	1.5%	15.38 [1.92; 45.45]
Doerge 1998	6	41	1.9%	2.4%	14.63 [5.57; 29.17]
Vohr2010	3	21	1.0%	1.9%	14.29 [3.05; 36.34]
Ullman 1999	5	40	1.6%	2.3%	12.50 [4.19; 26.80]
Osborne 2014	2	16	0.7%	1.5%	12.50 [1.55; 38.35]
Lund 1986	3	25	1.0%	1.9%	12.00 [2.55; 31.22]
Jako1995	3	25	1.0%	1.9%	12.00 [2.55; 31.22]
Takahashi 2012	3	25	1.0%	1.9%	12.00 [2.55; 31.22]
Kieny 1991	16	134	5.2%	2.9%	11.94 [6.98; 18.67]
Keeling 2016	25	214	8.2%	3.1%	11.68 [7.70; 16.76]
Argyriou 2024	25	256	8.4%	3.1%	9.77 [6.42; 14.08]
Sa 2007	1	12	0.3%	1.0%	8.33 [0.21; 38.48]
Yalamanchili 2004	1	13	0.3%	1.0%	7.69 [0.19; 36.03]
Neely 2015	8	115	2.8%	2.6%	6.96 [3.05; 13.25]
Shiomi 2016	2	31	0.7%	1.6%	6.45 [0.79; 21.42]
Leacche 2005	3	47	1.0%	1.9%	6.38 [1.34; 17.54]
Pasrij 2018	3	55	1.1%	2.0%	5.45 [1.14; 15.12]
F..dorov 2022	0	10	0.2%	0.6%	0.00 [0.00; 30.85]
Hartman 2015	4	96	1.4%	2.2%	4.17 [1.15; 10.33]
Rathore 2020	3	82	1.1%	2.0%	3.66 [0.76; 10.32]
Zarrabi 2013	1	30	0.4%	1.1%	3.33 [0.08; 17.22]
Azari 2015	1	30	0.4%	1.1%	3.33 [0.08; 17.22]
Lehnert 2012	1	33	0.4%	1.1%	3.03 [0.08; 15.76]
Edelman 2016	1	37	0.4%	1.1%	2.70 [0.07; 14.16]
Pasrij 2017	0	20	0.2%	0.6%	0.00 [0.00; 16.84]
Spagnolo 2006	0	21	0.2%	0.6%	0.00 [0.00; 16.11]
Keeling 2016	1	44	0.4%	1.1%	2.27 [0.06; 12.02]
Malekan 2012	0	26	0.2%	0.7%	0.00 [0.00; 13.23]
QiMin 2020	0	41	0.2%	0.7%	0.00 [0.00; 8.60]
**Total (common effect, 95% CI)**		**2340**	**100.0%**	.	**18.43 [16.69; 20.29]**
**Total (random effect, 95% CI)**			.	**100.0%**	**16.05 [12.95; 19.73]**

Heterogeneity: $Tau^2 = 0.4887$; $Chi^2 = 177.53$, df = 52 ($P < 0.01$); $I^2 = 71\%$

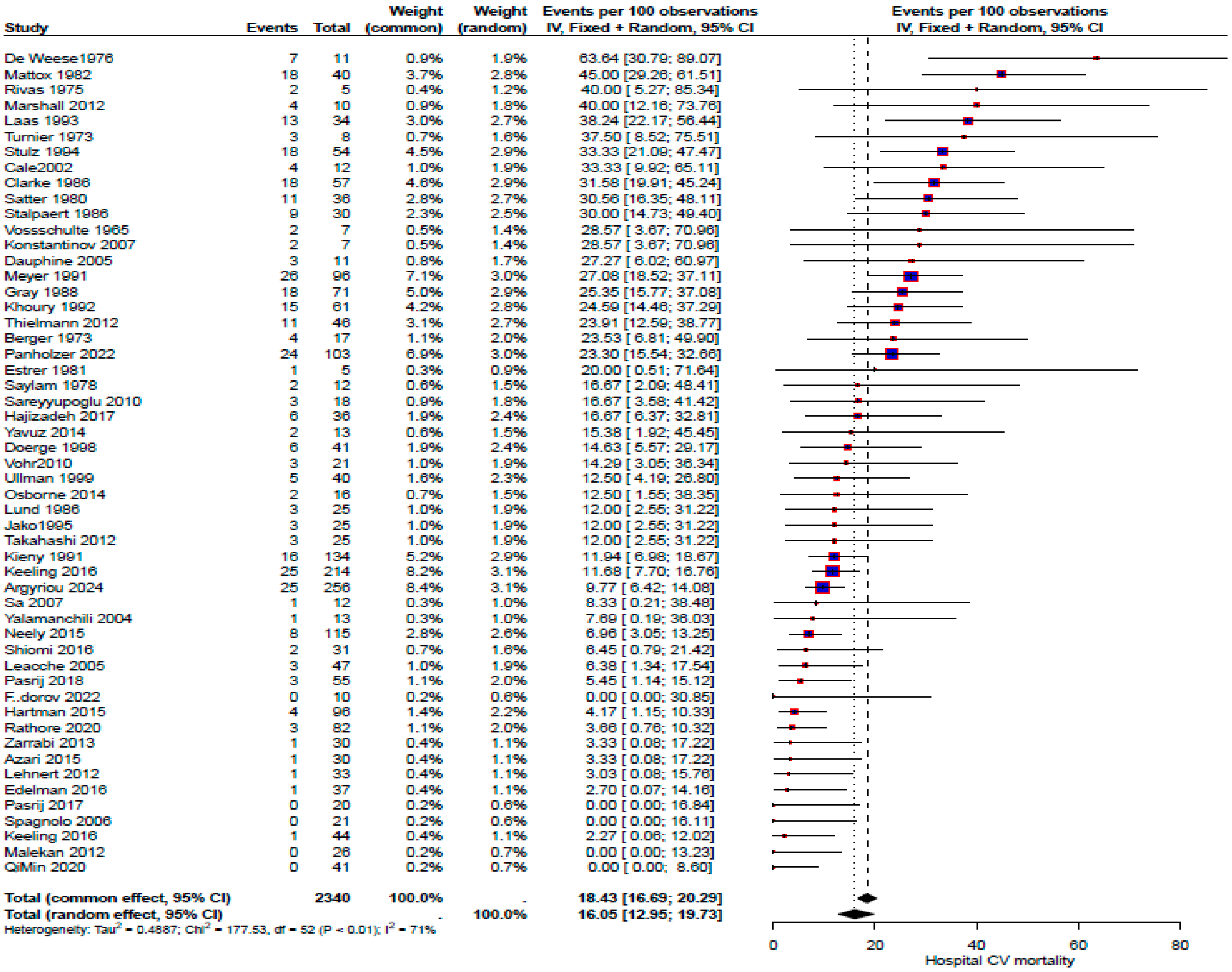

Figure S5. Forest plot for hospital CV mortality.

Study	Events	Total	Weight (common)	Weight (random)	Events per 100 observations IV, Fixed + Random, 95% CI
Digonnet 2007	8	21	5.9%	4.7%	38.10 [18.11; 61.56]
Ullman 1999	9	40	8.3%	5.3%	22.50 [10.84; 38.45]
Rivas 1975	1	5	1.0%	1.6%	20.00 [0.51; 71.64]
Ahmed 2008	3	15	2.9%	3.4%	20.00 [4.33; 48.09]
Amirghofran 2007	2	11	2.0%	2.7%	18.18 [2.28; 51.78]
Cale2002	2	12	2.0%	2.7%	16.67 [2.09; 48.41]
Doerge 1998	6	41	6.1%	4.8%	14.63 [5.57; 29.17]
Vossschulte 1965	1	7	1.0%	1.7%	14.29 [0.36; 57.87]
Meyer 1991	13	96	13.4%	6.0%	13.54 [7.41; 22.04]
Greelish 2011	2	15	2.1%	2.8%	13.33 [1.66; 40.46]
Jako1995	3	25	3.2%	3.6%	12.00 [2.55; 31.22]
Hajizadeh 2017	4	36	4.2%	4.1%	11.11 [3.11; 26.06]
Stulz 1994	5	54	5.4%	4.6%	9.26 [3.08; 20.30]
Lund 1986	2	25	2.2%	2.9%	8.00 [0.98; 26.03]
Yavuz 2014	1	13	1.1%	1.8%	7.69 [0.19; 36.03]
Mattox 1982	3	40	3.3%	3.7%	7.50 [1.57; 20.39]
Clarke 1986	4	57	4.4%	4.2%	7.02 [1.95; 17.00]
Shiomi 2016	2	31	2.2%	2.9%	6.45 [0.79; 21.42]
Laas 1993	2	34	2.2%	2.9%	5.88 [0.72; 19.68]
Sareyyupoglu 2010	1	18	1.1%	1.8%	5.56 [0.14; 27.29]
Rathore 2020	4	82	4.5%	4.2%	4.88 [1.34; 12.02]
F..dorov 2022	0	10	0.6%	1.1%	0.00 [0.00; 30.85]
Thielmann 2012	2	46	2.3%	3.0%	4.35 [0.53; 14.84]
Gray 1988	3	71	3.4%	3.7%	4.23 [0.88; 11.86]
Kieny 1991	5	134	5.7%	4.7%	3.73 [1.22; 8.49]
Stalpaert 1986	1	30	1.2%	1.9%	3.33 [0.08; 17.22]
Khoury 1992	2	61	2.3%	3.0%	3.28 [0.40; 11.35]
Edelman 2016	1	37	1.2%	1.9%	2.70 [0.07; 14.16]
Pasrij 2017	0	20	0.6%	1.1%	0.00 [0.00; 16.84]
Spagnolo 2006	0	21	0.6%	1.1%	0.00 [0.00; 16.11]
Malekan 2012	0	26	0.6%	1.1%	0.00 [0.00; 13.23]
Pasrij 2018	1	55	1.2%	1.9%	1.82 [0.05; 9.72]
Azari 2015	0	30	0.6%	1.1%	0.00 [0.00; 11.57]
QiMin 2020	0	41	0.6%	1.1%	0.00 [0.00; 8.60]
Keeling 2016	0	44	0.6%	1.1%	0.00 [0.00; 8.04]
**Total (common effect, 95% CI)**		**1304**	**100.0%**	.	**9.68 [7.96; 11.72]**
**Total (random effect, 95% CI)**			.	**100.0%**	**8.32 [6.22; 11.06]**

Heterogeneity: $Tau^2 = 0.3438$; $Chi^2 = 61.26$, df = 34 ($P < 0.01$); $I^2 = 44\%$

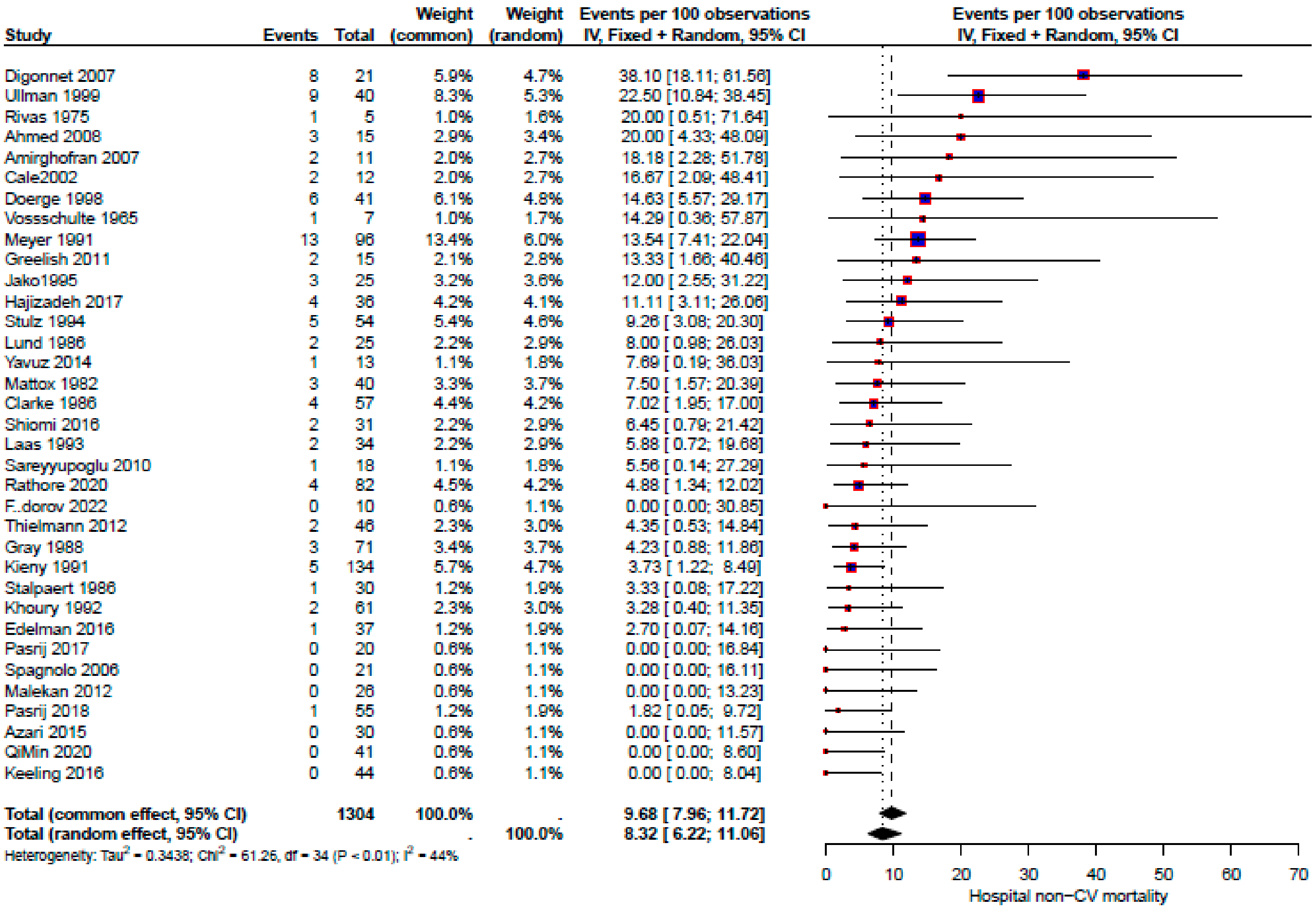

Figure S6. Forest plot of non-CV hospital mortality.

A.

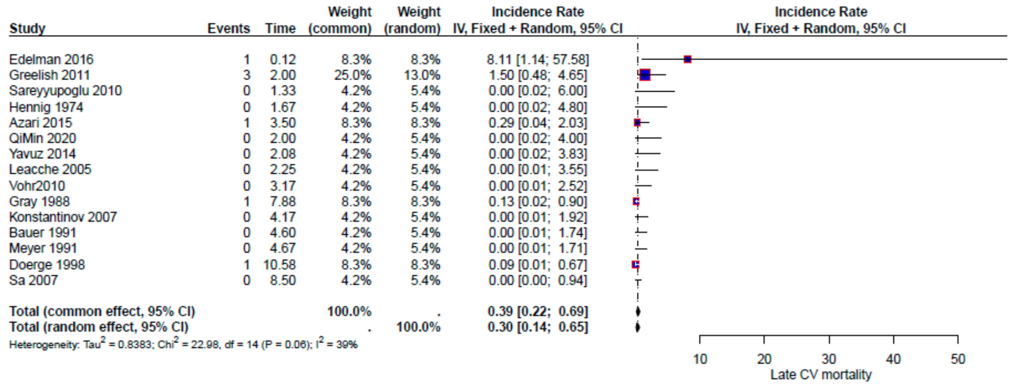

B.

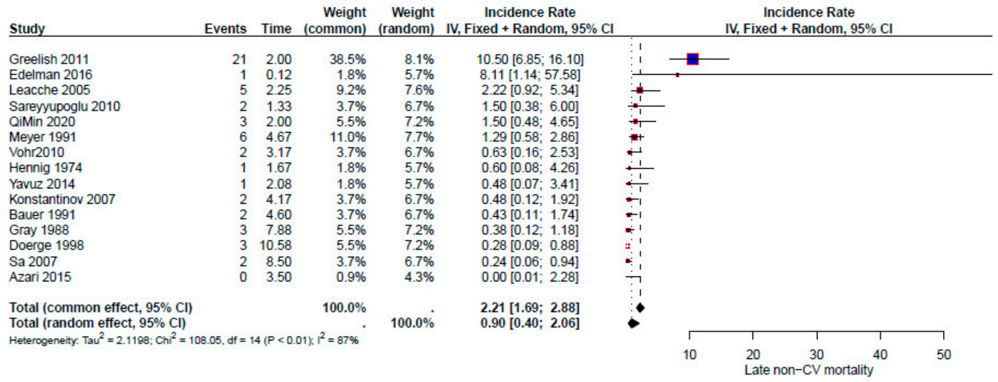

Figure S7. (**A**) Late cardiovascular (CV) mortality and (B) late non-CV mortality.

Study	Events	Total	Weight (common)	Weight (random)	Events per 100 observations IV, Fixed + Random, 95% CI
Greelish 2011	8	15	11.9%	10.1%	53.33 [26.59; 78.73]
Amirghofran 2007	4	11	8.1%	9.3%	36.36 [10.93; 69.21]
Sa 2007	3	12	7.2%	9.0%	25.00 [5.49; 57.19]
Ullman 1999	8	40	20.4%	11.0%	20.00 [9.05; 35.65]
Meyer 1991	8	96	23.4%	11.2%	8.33 [3.67; 15.76]
Yavuz 2014	1	13	2.9%	6.3%	7.69 [0.19; 36.03]
Bauer 1991	2	44	6.1%	8.5%	4.55 [0.56; 15.47]
Leacche 2005	2	47	6.1%	8.5%	4.26 [0.52; 14.54]
Jako1995	1	25	3.1%	6.5%	4.00 [0.10; 20.35]
Park 2019	1	27	3.1%	6.5%	3.70 [0.09; 18.97]
Khoury 1992	2	61	6.2%	8.6%	3.28 [0.40; 11.35]
Azari 2015	0	30	1.6%	4.4%	0.00 [0.00; 11.57]
**Total (common effect, 95% CI)**		**421**	**100.0%**	.	**13.34 [9.79; 17.93]**
**Total (random effect, 95% CI)**			.	**100.0%**	**10.62 [5.43; 19.74]**

Heterogeneity: $Tau^2 = 1.0926$; $Chi^2 = 40.59$, df = 11 ($P < 0.01$); $I^2 = 73\%$

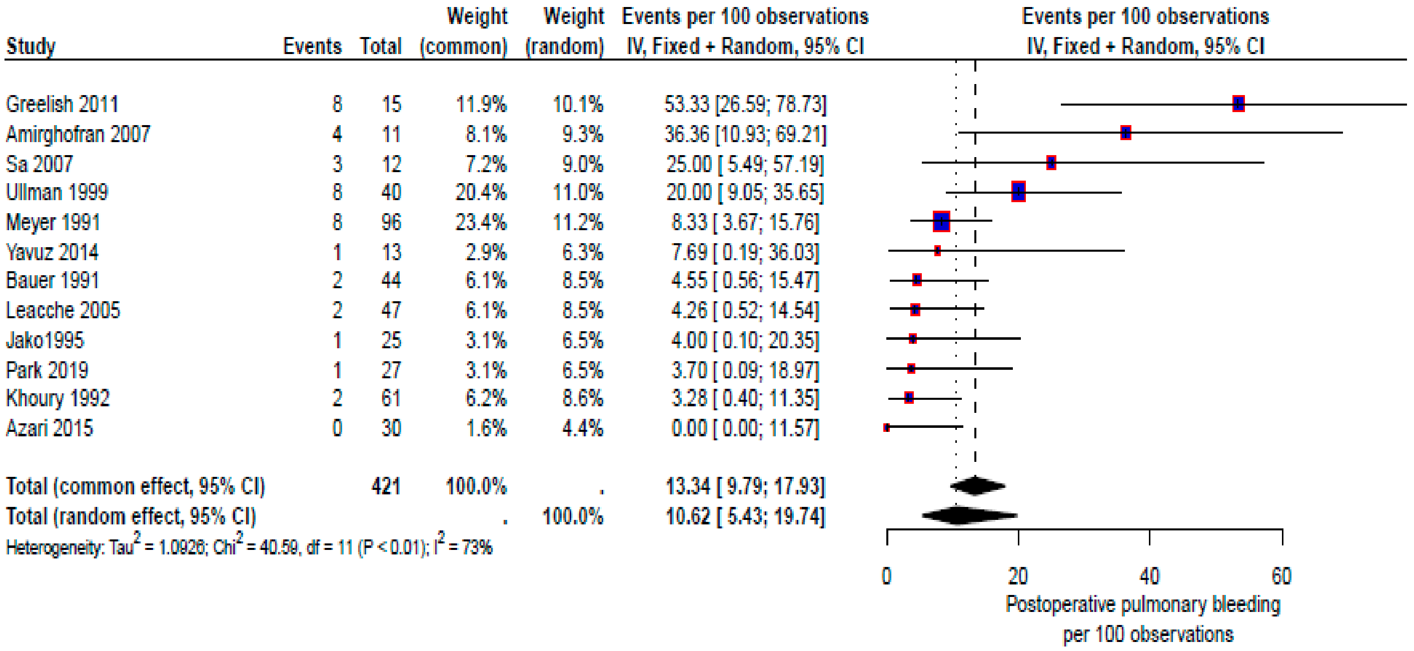

Figure S8. Forest plot of postoperative pulmonary bleeding.

Study	Events	Total	Weight (common)	Weight (random)	Events per 100 observations IV, Fixed + Random, 95% CI
Cale2002	2	12	11.4%	11.4%	16.67 [2.09; 48.41]
Clarke 1986	4	57	25.5%	25.5%	7.02 [1.95; 17.00]
Ullman 1999	2	40	13.0%	13.0%	5.00 [0.61; 16.92]
Lund 1986	1	25	6.6%	6.6%	4.00 [0.10; 20.35]
Takahashi 2012	1	25	6.6%	6.6%	4.00 [0.10; 20.35]
Hartman 2015	3	96	20.0%	20.0%	3.12 [0.65; 8.86]
Bauer 1991	1	44	6.7%	6.7%	2.27 [0.06; 12.02]
Azari 2015	0	30	3.4%	3.4%	0.00 [0.00; 11.57]
Gray 1988	1	71	6.8%	6.8%	1.41 [0.04; 7.60]
**Total (common effect, 95% CI)**		**400**	**100.0%**	.	**4.70 [2.86; 7.61]**
**Total (random effect, 95% CI)**			.	**100.0%**	**4.70 [2.86; 7.61]**

Heterogeneity: $Tau^2 = 0$; $Chi^2 = 7.19$, df = 8 ($P = 0.52$); $I^2 = 0\%$

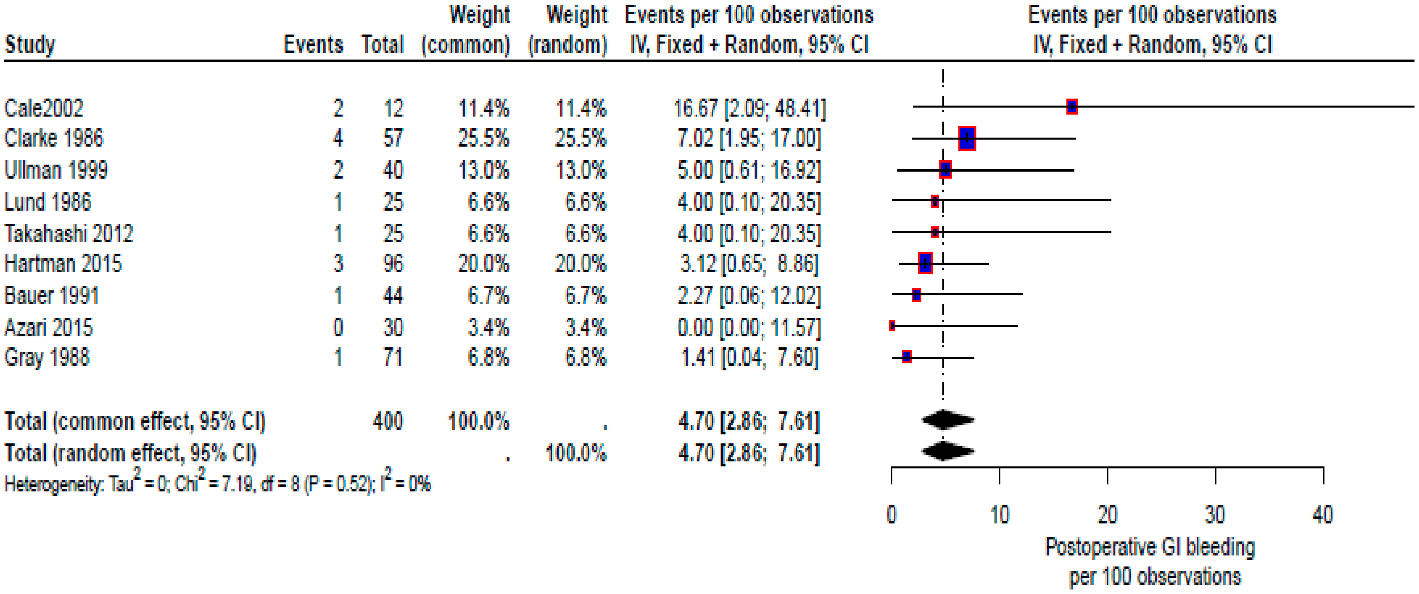

Figure S9. Forest plot of postoperative gastrointestinal (GI) bleeding.

Study	Events	Total	Weight (common)	Weight (random)	Events per 100 observations IV, Fixed + Random, 95% CI
Takahashi 2012	3	25	31.4%	29.7%	12.00 [2.55; 31.22]
Mkalaluh 2019	3	49	33.5%	31.2%	6.12 [1.28; 16.87]
Yavuz 2014	0	13	5.7%	6.6%	0.00 [0.00; 24.71]
Leacche 2005	1	47	11.7%	12.8%	2.13 [0.05; 11.29]
Azari 2015	0	30	5.9%	6.8%	0.00 [0.00; 11.57]
Gray 1988	1	71	11.7%	12.9%	1.41 [0.04; 7.60]
**Total (common effect, 95% CI)**		**235**	**100.0%**	**.**	**5.13 [2.67; 9.61]**
**Total (random effect, 95% CI)**		**.**		**100.0%**	**4.84 [2.36; 9.69]**

Heterogeneity: $Tau^2 = 0.1084$; $Chi^2 = 5.69$, df = 5 (P = 0.34); $I^2 = 12\%$

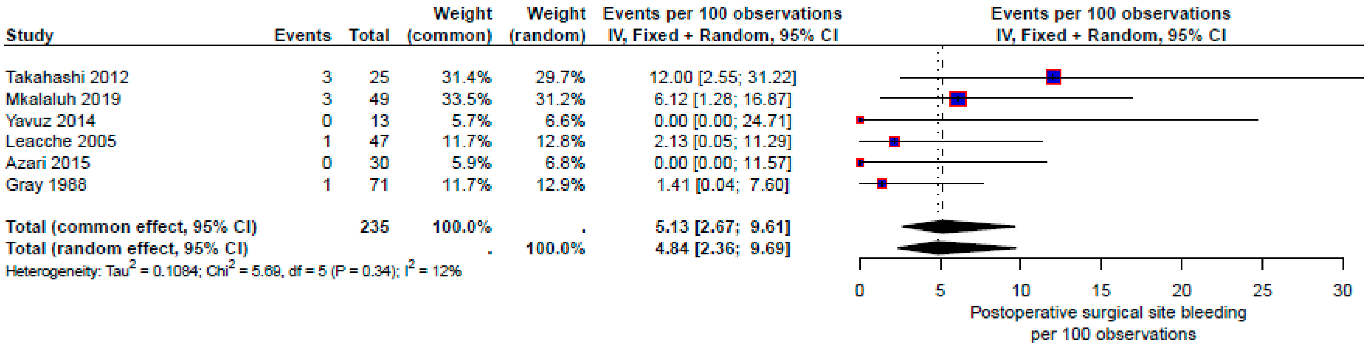

Figure S10. Forest plot bleeding at surgical site.

Study	Events	Total	Weight (common)	Weight (random)	Events per 100 observations IV, Fixed + Random, 95% CI
Cale2002	2	12	5.2%	7.1%	16.67 [2.09; 48.41]
Lund 1986	3	25	8.2%	9.8%	12.00 [2.55; 31.22]
Neely 2015	13	115	36.0%	18.9%	11.30 [6.16; 18.55]
Yavuz 2014	1	13	2.9%	4.5%	7.69 [0.19; 36.03]
Clarke 1986	4	57	11.6%	11.9%	7.02 [1.95; 17.00]
Bauer 1991	3	44	8.7%	10.1%	6.82 [1.43; 18.66]
Park 2019	1	27	3.0%	4.7%	3.70 [0.09; 18.97]
Hartman 2015	3	96	9.1%	10.3%	3.12 [0.65; 8.86]
Gray 1988	2	71	6.1%	8.0%	2.82 [0.34; 9.81]
Doerge 1998	1	41	3.0%	4.7%	2.44 [0.06; 12.86]
Azari 2015	0	30	1.5%	2.6%	0.00 [0.00; 11.57]
Keeling 2016	0	44	1.5%	2.6%	0.00 [0.00; 8.04]
Meyer 1991	1	96	3.1%	4.8%	1.04 [0.03; 5.67]
**Total (common effect, 95% CI)**		**671**	**100.0%**	**.**	**6.92 [5.00; 9.52]**
**Total (random effect, 95% CI)**		**.**		**100.0%**	**5.80 [3.68; 9.01]**

Heterogeneity: $Tau^2 = 0.2260$; $Chi^2 = 17.97$, df = 12 (P = 0.12); $I^2 = 33\%$

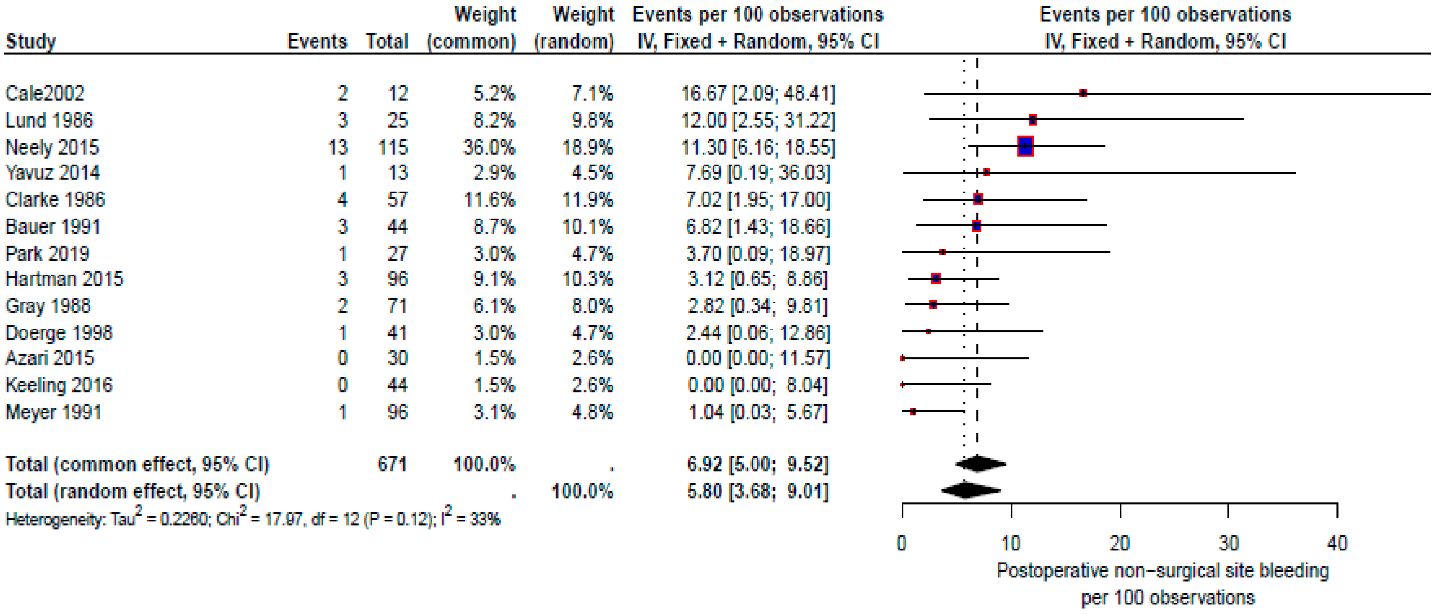

Figure S11. Forest plot bleeding at non-surgical site.

Study	Events	Total	Weight (common)	Weight (random)	Events per 100 observations IV, Fixed + Random, 95% CI
Wu 2013	4	25	6.0%	7.3%	16.00 [4.54; 36.08]
Vohr2010	3	21	4.6%	5.8%	14.29 [3.05; 36.34]
Panholzer 2022	12	103	19.0%	17.1%	11.65 [6.17; 19.47]
Dauphine 2005	1	11	1.6%	2.2%	9.09 [0.23; 41.28]
Cale2002	1	12	1.6%	2.2%	8.33 [0.21; 38.48]
Argyriou 2024	19	256	31.5%	22.8%	7.42 [4.53; 11.35]
Leacche 2005	3	47	5.0%	6.2%	6.38 [1.34; 17.54]
Mkalaluh 2019	3	49	5.0%	6.3%	6.12 [1.28; 16.87]
Neely 2015	7	115	11.8%	12.4%	6.09 [2.48; 12.14]
Meyer 1991	4	96	6.9%	8.1%	4.17 [1.15; 10.33]
Jako1995	1	25	1.7%	2.3%	4.00 [0.10; 20.35]
Pasrij 2018	1	55	1.8%	2.4%	1.82 [0.05; 9.72]
Khoury 1992	1	61	1.8%	2.4%	1.64 [0.04; 8.80]
Azari 2015	0	30	0.9%	1.2%	0.00 [0.00; 11.57]
Keeling 2016	0	44	0.9%	1.2%	0.00 [0.00; 8.04]
**Total (common effect, 95% CI)**		**950**	**100.0%**	**.**	**7.45 [5.83; 9.47]**
**Total (random effect, 95% CI)**			**.**	**100.0%**	**7.20 [5.36; 9.60]**

Heterogeneity: $Tau^2 = 0.0557$; $Chi^2 = 16.60$, df = 14 ($P = 0.28$); $I^2 = 16\%$

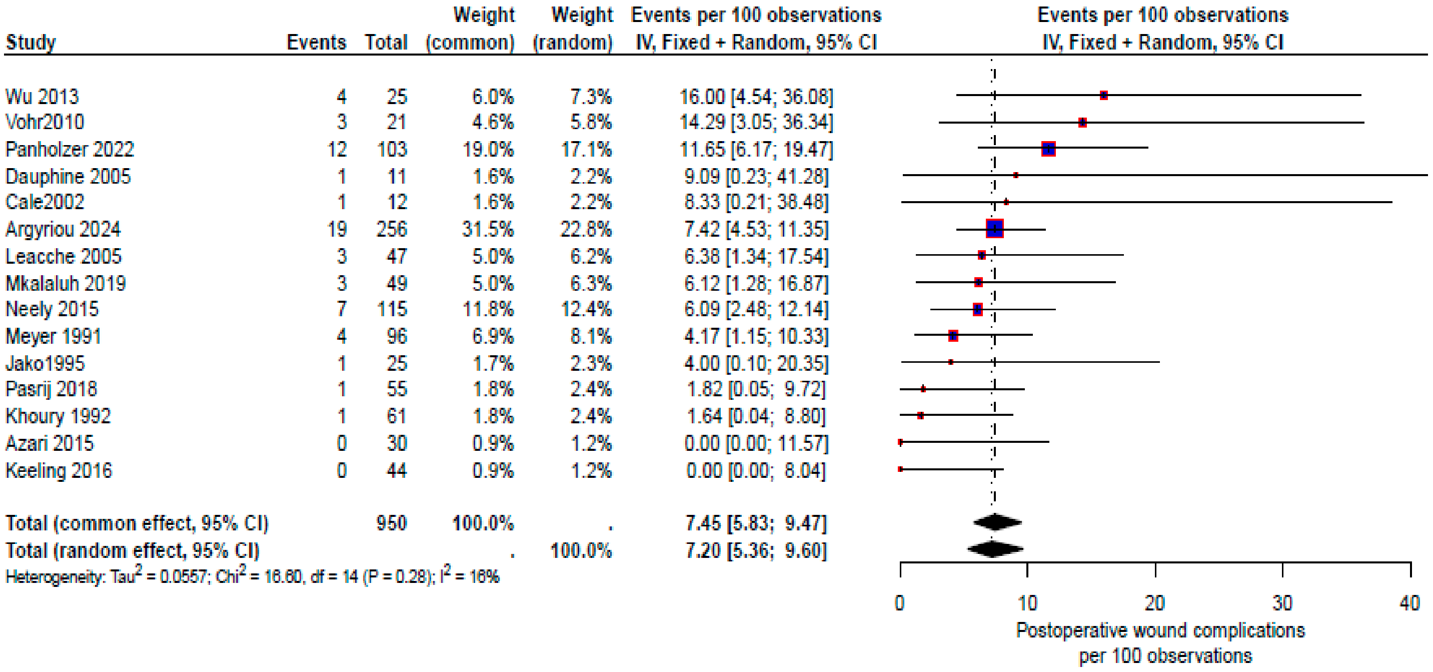

Figure S12. Wound complications.

Table S1. Intraoperative criteria of included studies.

Author, Year	Systemic Thrombolytics Contraindication percent	Preoperative Cardiac Arrest percent	RV Dysfunction percent	Need for Inotropes or Vasopressors percent	Preoperative Mechanical Ventilation, CPB, or ECC percent	Systemic Thrombolysis percent	Use of Cardiopulmonary Bypass percent	Use of Myocardial Protective Techniques percent	Use of Intraoperative Hypothermia percent	Use of Aortic Cross Clamping percent	Therapies available in the centers performed analysis	Pregnant women percent	SPE indications	IVC filter percent
Ahmed 2008	20.00		80.00	40.00	53.30	26.70	93.30	6.70		6.70	NR	NR	RVF	100
Alqahtani 2019		13.00				7.00					NR	excluded	NR	NR
Amirghofran 2007		27.30					100.00			0.00	NR	NR	NR	NR
Argyriou 2024			48.00				100.00			100.00	NR	NR	HF 48%, cardiac shock 37.5	NR
Azari 2015	30.00	20.00	100.00		20.00	26.66	100.00	100.00	100.00	100.00	NR	NR	Fibrinolytics CI30%, Fibrinolysis failure 26.7%, CA 20%,, PFO 20%, RT atrial clot 10%, cardiac shock 10%	NR
Barrett 2010	100.00	44.40				0.00					embolectomy 17.6%., thrombolysis 19.6, heparin 27.45%	NR	NR	NR
Bauer 1991	15.90	34.10		29.50			100.00	100.00	0.00		NR	NR	central PE and shock	100
Bennett 2015			90.00	12.50	12.50		12.50				NR	NR	NR	NR
Berger 1973							100.00				NR	NR	Occlusion >50%, saddle embolism	NR
Biglioli 1991						54.50					thrombolytics 60%	NR	cardiogenic shock 20%, massive PE 13.33%, RT atrial embolism 6.66%	NR
Bottzauw 1981	30.40	69.60	52.20			0.00	100.00				NR	NR	NR	NR
Boulafendis 1991		31.30		100.00	100.00	0.00	100.00				NR	NR	cardiogenic shock 100%	NR
Cale 2002	25.00	41.70		100.00			100.00			0.00	NR	NR	NR	NR
Clarke 1986		34.50					0.00	100.00	0.00		NR	NR	NR	NR
Dauphine 2005	45.50	36.40		36.40		27.30	100.00				NR	NR	NR	100
De Weese1976			45.50		45.50		90.90				NR	NR	NR	NR
DiChiacchio 2019			100.00		100.00		100.00				NR	NR	NR	NR

Digonnet 2007	4.80	28.60	100.00		4.80		100.00		0.00	33.30	SE, catheter retrieval, thrombolytics	NR	RVF	100
Doerge 1998	9.80	34.10		80.50	58.50	14.60	100.00	90.20	90.20	90.20	anticoagulants	NR	cardiogenic shock	NR
Dohle 2018		23.00				6.30	100.00			59.00	anticoagulation	NR	CA, shock, failed fibrinolysis, bilateral thrombus	NR
Edelman 2016		2.70	54.00		2.70	8.10	100.00		0.00		NR	NR	NR	NR
Estrer 1981		60.00		80.00			100.00		0.00		NR	NR	NR	NR
Fedorov 2022			100.00				100.00	100.00			NR	NR	cardiogenic shock, MOF	NR
Glassford 1981		50.00					100.00				NR	NR	NR	NR
Gray 1988	29.60	39.40				2.80	90.10				anticoagulation	NR	hemodynamically compromise 100%	NR
Greelish 2011	26.70	13.30	100.00		6.70		100.00		0.00		anticoagulation	NR	RVF	100%
Hajizadeh 2017		8.00	69.40	5.00			100.00				NR	NR	NR	NR
Hartman 2015	44.80	0.00	100.00	25.00		5.20	100.00	0.00	0.00		NR	NR	RVF	NR
Hennig 1974							0.00				NR	NR	NR	NR
Jako1995						24.00	100.00		100.00		NR	NR	RVF	100
Jaumin 1986											NR	NR	NR	NR
Keeling 2016		28.00					100.00			37.40	NR	NR	RVF	NR
Keeling 2016		0.00	100.00	20.50			100.00	100.00	100.00	100.00	NR	NR	RVF	100
Khoury 1992		27.90		63.90		11.50	100.00			0.00	NR	NR	NR	NR
Kieny 1991		17.20		26.10		17.20	74.40				NR	NR	Cardiac shock 25.37, arrest 17.1	NR
Konstantinov 2007		85.70	28.60		14.30	0.00	100.00		100.00	0.00	NR	NR	NR	NR
Laas 1993							100.00	76.50			NR	NR	NR	NR
Leacche 2005	44.70	12.80	95.20			8.50	100.00		0.00		NR	NR	contraindication of thrombolysis 45%, failed treatment 10%, RVF 32%	100
Lehnert 2012	39.40	3.00	97.00	42.40	18.20	18.20	100.00		0.00	21.20	NR	NR	CI of thrombolysis 13.13%, cardiac shock 18%	NR
Lund 1986		28.00	44.00				100.00			0.00	thrombolysis, anticoagulation	NR	RVF	NR
Malekan 2012			73.10	7.70	3.80		100.00		100.00		NR	NR	RVF	NR
Marshall 2012	100.00	60.00	100.00			0.00	90.00			10.00	NR	NR	Syncope 70%, CA 40%	NR
Mattox 1982		87.20			87.20		100.00	0.00		100.00	NR	NR	RVF	100
Meyer 1991	10.40	25.00	88.50	81.30		31.30	100.00	100.00	0.00	0.00	throbolysis	NR	shock	39.58
Meyns 1992		40.00				3.30	100.00				thrombolysis	NR	NR	NR

Minakawa 2018		27.60	50.00	11.50	27.05		100.00			58.00	NR	NR	Shock 50%	NR
Mkalaluh 2019		14.00	47.00		14.00	10.00	100.00	100.00	100.00	55.10	throm-bolysis 10%	NR	CA 14%, shock 47%	NR
Neely 2015	42.80	10.67	69.00			57.40	100.00		0.00	0.00	NR	RVF	NR	NR
Newcom2022		13.00	93.75								NR	NR	NR	NR
Osborne 2014		6.70	73.30				100.00	0.00			NR	NR	NR	NR
Panholzer 2022	56.30	29.10				12.60	14.50				throm-bolysis 12.6%	NR	NR	NR
Park 2019	7.40	7.40	100.00	3.70	7.40		100.00	100.00	100.00	100.00	NR	NR	RVF	NR
Pasrij 2017	25.00		100.00				100.00	75.00	75.00	75.00	NR	NR	RVF	100
Pasrij 2018	16.36	16.00	100.00	16.00	11.00		100.00	100.00	0.00		NR	NR	RVF	100
QiMin 2020	36.60	7.31	82.93			4.87	100.00			24.40	throm-bolysis, anticoag-ulation	NR	CA 4.87%	NR
Rathore 2020		14.60	98.00	54.80	17.24	25.60	100.00				NR	NR	RVF, hyper-lactemia, shock	NR
Rivas 1975							100.00				NR	NR	NR	NR
Sa 2007	33.30		66.70			58.30	58.30	58.30	58.30		NR	NR	NR	100
Salehi 2013			90.00	18.80							NR	NR	NR	NR
Sareyyupoglu 2010	72.20	38.90	72.20	50.00	50.00		100.00		0.00	5.60	NR	NR	Shock67 %, RVF 28%	NR
Satter 1980	100.00						100.00				NR	NR	NR	NR
Saylam 1978		12.50		100.00	12.50		100.00	100.00			NR	NR	NR	NR
Shiomi 2016		25.80	67.60	67.70	22.60		100.00			0.00	anticoag-ulation	NR	CA 25.5%, RVF100 %	NR
Spagnolo 2006		23.80	100.00	90.50			100.00	100.00	0.00	100.00	NR	NR	NR	NR
Stalpaert 1986							100.00		100.00	100.00	NR	NR	NR	NR
Stulz 1994		62.00		96.00	2.00	2.00	42.00				NR	NR	RVF	NR
Takahashi 2012	16.70	45.80	100.00		66.70	12.50	100.00	29.20	100.00	87.50	NR	NR	Shock, RVF	NR
Taniguchi 2012		9.40	9.40		31.30	25.00					NR	NR	RVF	100
Thielmann 2012		26.10	82.60	63.00	65.20	15.20	100.00	34.80			NR	NR	shock	NR
Turnier 1973		50.00					100.00				NR	NR	NR	NR
Ullman 1999		37.50		32.50			100.00	15.00		32.50	NR	NR	NR	NR
Vohr2010	47.60	42.90	100.00	66.70		52.40	81.00	14.30	19.00		NR	NR	NR	NR
Vossschulte 1965		14.30									NR	NR	NR	NR
Wu 2013	96.00	32.00	96.00			4.00	100.00	60.00	88.00		NR	NR	NR	NR
Yalamanchili 2004		15.40	100.00	30.80	46.20		100.00		0.00	0.00	NR	NR	NR	NR
Yavuz 2014		30.80	100.00	100.00	30.80	0.00	100.00			69.20	NR	NR	NR	NR
Zarrabi 2013											NR	NR	NR	100
Zielinski 2023	20.00			15.00			100.00	100.00	100.00	100.00	NR	NR	NR	NR

CA: cardiac arrest, CI: contraindication, IVC: inferior vena cava, NR: not reported, RVF: right ventricular failure,

Table S2. Newcastle Ottawa scale for quality assessment of included studies.

First Author, Year	Representativeness	Selection of Non-Exposed Cohort	Ascertainment of Exposure	Demonstration that Outcome Was Not Present at Start of Study	Comparability of Cohorts	Assessment of Outcome	Length of Follow-up	Adequacy of Follow-up
Ahmed 2008	**	NR	*	*	NR	*	*	*
Alqahtani 2019	**	NR	*	*	NR	NR	*	*
Amirghofran 2007	**	NR	*	*	NR	*	*	*
Argyriou 2024	**	NR	*	*	NR	**	*	*
Azari 2015	**	NR	**	*	NR	*	*	*
Barrett 2010	**	NR	*	*	NR	*	*	*
Bauer 1991	**	NR	**	*	NR	*	*	*
Bennett 2015	**	NR	**	*	NR	*	*	*
Berger 1973	**	NR	*	*	NR	*	*	*
Biglioli 1991	**	NR	*	*	NR	*	*	*
Bottzauw 1981	**	NR	*	*	NR	*	*	*
Boulafendis 1991	**	NR	*	*	NR	*	*	**
Cale2002	**	NR	*	*	NR	*	*	*
Clarke 1986	**	NR	*	*	NR	*	*	*
Dauphine 2005	**	NR	*	*	NR	*	*	**
De Weese1976	**	NR	*	*	NR	*	*	*
DiChiacchio 2019	**	NR	*	*	NR	*	*	*
Digonnet 2007	**	NR	*	*	NR	*	*	**
Doerge 1998	**	NR	**	*	NR	**	*	**
Dohle 2018	**	NR	*	*	NR	**	*	*
Edelman 2016	**	NR	**	*	NR	**	*	**
Estrer1981	**	NR	**	*	NR	*	*	*
Fedorov 2022	**	NR	*	*	NR	**	*	*
Glassford 1981	**	NR	**	*	NR	*	*	*
Gray 1988	**	NR	*	*	NR	*	*	**
Greelish 2011	**	NR	*	*	NR	**	*	**
Hajizadeh2017	**	NR	**	*	NR	*	**	**
Hartman 2015	**	NR	**	*	NR	**	*	**
Hennig 1974	**	NR	*	*	NR	*	*	**
Jako1995	**	NR	*	*	NR	**	*	**
Jaumin 1986	**	NR	*	*	NR	*	*	*
Keeling 2016 (A)	**	NR	**	*	NR	**	*	**
Keeling 2016 (B)	**	NR	**	*	NR	**	**	**
Khoury 1992	**	NR	*	*	NR	*	*	*
Kieny 1991	**	NR	*	*	NR	*	*	*
Konstantinov 2007	**	NR	*	*	NR	*	*	**
Laas 1993	**	NR	*	*	NR	*	*	**
Leacche 2005	**	NR	*	*	NR	**	*	**
Lehnert 2012	**	NR	*	*	NR	**	*	**
Lund 1986	**	NR	*	*	NR	*	*	*
Malekan 2012	**	NR	*	*	NR	*	*	**
Marshall 2012	**	NR	*	*	NR	*	*	**
Mattox 1982	**	NR	*	*	NR	*	*	*
Meyer 1991	**	NR	**	*	NR	**	*	**
Meyns 1992	**	NR	*	*	NR	*	*	**
Minakawa 2018	**	NR	*	*	NR	*	**	**
Mkalaluh 2019	**	NR	*	*	NR	**	***	***
Neely 2015	**	NR	**	*	NR	*	*	**
Newcom2022	**	NR	*	*	NR	**	*	*
Osborne 2014	**	NR	*	*	NR	**	*	*
Panholzer 2022	**	NR	*	*	NR	**	*	*
Park 2019	**	NR	*	*	NR	**	*	**
Pasrij 2017	**	NR	*	*	NR	*	*	*
Pasrij2018	**	NR	**	*	NR	**	*	*
QiMin 2020	**	NR	*	*	NR	**	*	*
Rathore 2020	**	NR	*	*	NR	**	*	*
Rivas 1975	**	NR	*	*	NR	*	*	*
Sa 2007	**	NR	**	*	NR	*	*	**
Salehi 2013	**	NR	*	*	NR	*	*	**
Sareyyupoglu 2010	**	NR	*	*	NR	*	*	**
Satter 1980	**	NR	*	*	NR	*	*	*
Saylam 1978	**	NR	*	*	NR	*	*	*
Shiomi 2016	**	NR	*	*	NR	*	*	**
Spagnolo 2006	**	NR	**	*	NR	*	*	*

Stalpaert 1986	**	NR	*	*	NR	*	*	*
Stulz 1994	**	NR	*	*	NR	*	*	*
Takahashi 2012	**	NR	**	*	NR	**	*	**
Taniguchi 2012	**	NR	*	*	NR	*	*	**
Thielmann 2012	**	NR	*	*	NR	*	*	**
Turnier 1973	**	NR	*	*	NR	*	*	*
Ullman 1999	**	NR	*	*	NR	*	*	**
Vohr 2010	**	NR	**	*	NR	**	*	**
Vossschulte 1965	**	NR	*	*	NR	*	*	*
Wu 2013	**	NR	*	*	NR	**	*	**
Yalamanchili 2004	**	NR	*	*	NR	*	*	*
Yavuz 2014	**	NR	*	*	NR	**	*	**
Zarrabi 2013	**	NR	*	*	NR	**	*	**
Zielinski 2023	**	NR	**	*	NR	**	*	*

NR: not-reported
